# Molecular effects of dADD1 misexpression in chromatin organization and transcription

**DOI:** 10.1186/s12860-020-00257-2

**Published:** 2020-03-23

**Authors:** Silvia Meyer-Nava, Amada Torres, Mario Zurita, Viviana Valadez-Graham

**Affiliations:** Instituto de Biotecnología. Universidad Nacional Autónoma de México, Campus Morelos, Av. Universidad 2001, C.P, 62210 Cuernavaca, Morelos Mexico

**Keywords:** Heterochromatin, dADD1, ATRX, dXNP

## Abstract

**Background:**

dADD1 and dXNP proteins are the orthologs in *Drosophila melanogaster* of the ADD and SNF2 domains, respectively, of the ATRX vertebrate’s chromatin remodeler, they suppress position effect variegation phenotypes and participate in heterochromatin maintenance.

**Results:**

We performed a search in human cancer databases and found that ATRX protein levels were elevated in more than 4.4% of the samples analyzed. Using the *Drosophila* model, we addressed the effects of over and under-expression of dADD1 proteins in polytene cells. Elevated levels of dADD1 in fly tissues caused different phenotypes, such as chromocenter disruption and loss of banding pattern at the chromosome arms. Analyses of the heterochromatin maintenance protein HP1a, the dXNP ATPase and the histone post-translational modification H3K9me3 revealed changes in their chromatin localization accompanied by mild transcriptional defects of genes embedded in heterochromatic regions. Furthermore, the expression of heterochromatin embedded genes in null *dadd1* organisms is lower than in the wild-type conditions.

**Conclusion:**

These data indicate that dADD1 overexpression induces chromatin changes, probably affecting the stoichiometry of HP1a containing complexes that lead to transcriptional and architectural changes. Our results place dADD1 proteins as important players in the maintenance of chromatin architecture and heterochromatic gene expression.

## Background

The major factors involved in chromatin dynamics are ATP-dependent chromatin remodeling complexes, which contain an ATPase catalytic subunit, which provides the energy necessary for their function. One of these ATPases is ATRX, first described as a putative member of the helicase superfamily due to its homology with RAD54 that has been implicated in nucleotide excision repair and transcription [1, 2]. Mutations in the human gene are the main cause of a syndrome that includes alpha thalassemia, profound developmental delay, mental retardation, genital abnormalities, and facial dimorphism, among other manifestations [2]. The *ATRX* gene is highly conserved through eukaryotic evolution; for example, mouse and human proteins have 87% homology [3], whereas invertebrates like *Drosophila melanogaster* have 66% [4].

The human mutations usually generate a change in protein functionality and mostly fall into the helicase-ATPase domain in the carboxy terminus, or the ADD motif (named after the three proteins that carry it, **A**TRX-**D**NMT3-**D**NMT3L), composed of a PHD and a GATA-like zinc fingers, which recognize the H3K9me3 and the unmethylated H3K4 combination of histone marks [5]. This domain directs the protein mainly to pericentric heterochromatin [6]. Although there has also been described that the ATRX PxVxL motif can target ATRX through HP1a, and mutations in this motif [7] reduce the localization of ATRX in the heterochromatin [6].

Mutations that affect the function of ATRX have recently been proposed as markers of poor survival in soft tissue sarcomas [8]. It has been highlighted that inactivating mutations in the ATRX/DAXX/H.3.3 complex in cells displaying alternative lengthening of telomeres (ALT) phenotype, including pancreatic neuroendocrine tumors [9], glioblastoma multiform, oligodendrogliomas, medulloblastomas [10] and neuroblastomas [11], support the potential role of ATRX as a tumor suppressor. Endogenous expression of ATRX suppressed the ALT pathway on bone osteosarcoma epithelial human cells [12]. Also, in a murine model of ATRX overexpression, several phenotypes were observed, such as neural tube defects, growth retardation, high mortality, and problems in locomotion and behavior in organisms that survived postnatally [13].

In *Drosophila*, the two main domains of the human ATRX protein are encoded by two different genes *dxnp* and *dadd1*. dXNP proteins conserve a helicase/ATPase domain but lack the ADD domain. The *dadd1* gene encodes three ADD harboring isoforms generated by alternative splicing. Our group and others have shown a physical interaction of these proteins with HP1a and also their localization to heterochromatic regions [14, 15]. We found that in somatic cells, mutations in *dadd1* affect chromosome stability, induce telomeric defects in the fly such as telomeric fusions, and loss of retrotransposon silencing. Lack of dADD1 caused delocalization of HP1a protein from the telomeres, with slight disturbances at other chromosomal sites [16].

Genomic instability is an indication of cancer, and it is supposed to promote tumorigenesis in pre-cancerous lesions, as well as karyotypic diversity during cancer progression. Some of the hypothesis identifies two potential pathways, the loss of tumor suppressor gene functions and/or activation of oncogenes [17].

There have been studies linking different levels of expression of ATRX as drivers of specific phenotypes that give rise to disease and cancer [13, 18, 19]. In the present study, we searched human somatic cancer databases and found that the *ATRX* gene is overexpressed in a wide variety of human cancers. Using a *Drosophila* model of ATRX, we modified the expression levels of dADD1 proteins and evaluated the effects of this overexpression in polytene cells. When dADD1 proteins have higher than wild-type levels, the polytene chromosomes lose compaction and banding pattern. HP1a protein delocalizes and acquires a different distribution within the cell nucleus. To address the roles of the dADD1a and b protein isoforms, we modified the levels of the proteins independently, and overexpression of either isoform leads to changes in the chromatin localization of HP1a, dXNP and also H3K9me3 with differences in the expression of heterochromatin and some euchromatin embedded genes.

We conclude that overexpression of dADD1 proteins titrates the levels of heterochromatin formation proteins leading to chromatin architecture loss, chromosomal instability, and organism death. Our results are discussed in the context of the cellular effects of dADD1 proteins, which are essential for global chromosome stability.

## Results

### ATRX expression levels in human cancers

Mutations that affect the function of ATRX have been associated with several types of cancers, including glioblastoma and pancreatic cancer [20, 21] and ATRX aberrant expression has been recently proposed as a marker of poor survival in soft tissue sarcomas [8]. On the contrary, higher levels of ATRX protein have not described in human cancers. We decided to analyze the levels of ATRX transcripts in a variety of human somatic cancers using the COSMIC (Catalogue Of Somatic Mutations In Cancer) and THPA (The Human Protein Atlas), database in detail [22, 23]. This search found that ATRX is expressed in all types of cells reviewed on THPA database with high TPM (Transcripts Per Million) values, principally in tissues like parathyroid and thyroid glands, cerebral cortex and endometrium, the thresholds used to categorize over- and under-expression from normal levels is explained in the database. The protein is also present in almost all types of human tissues [23]. According to the Genomic Data Commons (GDC) database, which is a general database, ATRX is one of the most frequently mutated genes associated with cancer, alterations in its expression are present in 7.79% of the reported cases (795/10,202 cases). Variations in gene copy number are only reported in a low number of cases with 1.76% (184/10473 cases) for gain and 1.59% for loss (167/10,473) on GDC and 0.12% (7/5686 samples) for gain and 0.81% (46/5686 samples) for loss on COSMIC, which is a manually curated database.

Advanced filtered query on COSMIC reported 174 (overexpressing) and 39 (underexpressing) cases that had modified levels of ATRX, corresponding to 4.45 and 0.99% of the total of cases registered (*n* = 3910), respectively. Cases with no previous history of treatment to rule out possible secondary effects from any treatment were selected, which gave us a total of 144 overexpressing and 33 under-expressing cases. Additional File 1a shows the distribution of ATRX overexpression in different types of tumors like lung (19%) and breast (26%) tumors are the most abundant type of cancers with alterations in the expression; we did not find any records of central nervous system tumors represented in this group. The mean overexpression revealed a fold change ranking between 3 to 5.5 in comparison to the control tissue (data not shown). Additional File 1b shows the types of tumors associated with a lesser amount of ATRX expression, like lung (38%), upper aerodigestive tract (18%) and Central Nervous System (CNS) with 5%. Under-expression is not correlated to changes in the gene copy number, indicating that, as the overexpression cases, these changes could result from other molecular events such as gene repression or perhaps mutations in regulatory and coding sequences. Examples of Breast and Lung tissues from the THPA database are shown in the Additional File 1; ATRX immunohistochemistry was performed with the same antibody (Santa Cruz) in healthy breast tissue (Additional File 1c) where we can see nuclear localization in the gland cells. Additional Fig. 1d shows a breast cancer sample with low levels of ATRX and in Additional File 1e and 1f high levels, we can see high levels of ATRX protein in breast cancer samples. Lung tissue immunostaining with the same antibody is shown in g and h; these samples show low ATRX levels. Additional Fig. 1i and j are lung carcinoma examples in which ATRX is overexpressed with strong staining and nuclear localization. These data associate ATRX overexpression to several types of somatic tumors, therefore more in-depth cellular analyses of the molecular effects of ATRX overexpression are essential to study its role as one of the factors that may lead to the appearance or maintenance of a transformed tumor phenotype.

### Misexpression of dADD1 disrupts chromatin structure

Human ATRX has two important domains that cooperate to exert its functions, the ADD domain, which recognizes the H3K9me3 and H3K4 unmethylated histone mark, which directs this protein to heterochromatic regions, and the SNF2 domain which is necessary for the correct H3.3 exchange by DAXX [24]. In insects, these two domains are separated and encoded by different genes, *dadd1* encodes orthologues to the amino ADD domain of ATRX and *dxnp* encodes proteins orthologues to the SNF2 domain [15].

**Fig. 1:**
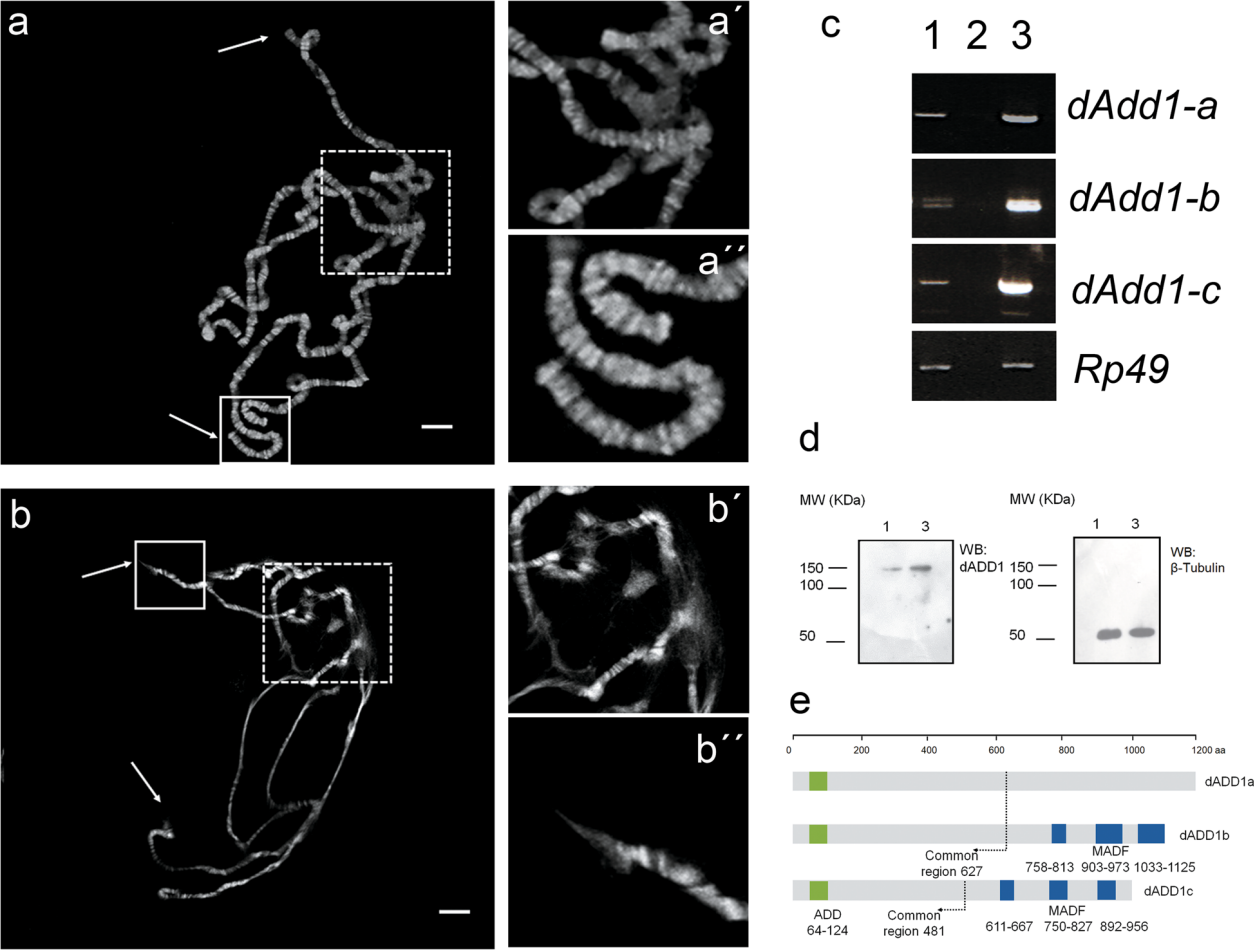
Modification of dADD1 expression levels results in chromosomal disorganization. **a** Polytene chromosome squash from a wild-type organism. DNA was stained with DAPI (shown in gray), arrows point to telomeric regions, the chromocenter is shown within the dashed box a’) magnification of the chromocenter, a”) magnification of the telomeres. **b** Polytene chromosome squash from salivary glands in which over-expression of dADD1 isoform was performed using the UAS-GAL4 system, as in “a” arrows point to telomeric regions and the chromocenter is in the dashed box, b’) magnification of the chromocenter, b”) magnification of a telomeric region. Scale bar 20 μm. **c** Semi-quantitative analysis of the transcript levels of dADD1 isoforms in salivary glands. Lanes in the gel correspond to 1) wild-type, 2) H_2_O, 3) *UAS-dadd1; Sgs3-GAL4* genotypes. *Rp49* transcript was used as a control. Note that all transcripts isoforms are overexpressed (compare lanes 1 to 3). **d** Western blot from total protein extracts from salivary glands where pan-dADD1 and anti-β tubulin antibodies were used. Lanes correspond to 1) wild-type and 3) *UAS-dadd1; Sgs3-GAL4,* molecular weight markers are shown on the left side of the panels. On the right side of the panels, the antibodies used for each membrane are specified. **e** Schematic representation of the dADD1 protein isoforms. The ADD domain shared by all isoforms is shown in green; a dashed line delimits the common region. MADF domains of the dADD1b and c isoforms are shown in blue. dADD1a is 1199 aa long (130 kDa), dADD1b is 1125 aa (127 kDa) and dADD1c is 979 aa (112 kDa)

The *dadd1* gene encodes three alternative spliced isoforms (see Fig. 1e), in a previous publication we showed that dADD1a tethers HP1a to the telomeres, and in a *Drosophila* line that lacks the *dadd1* gene, a set of telomeric retrotransposons (called the HTT array) which participate in telomeric maintenance and are normally silenced in somatic cells, are expressed. This led to longer telomeres and chromosomal aberrations [16]. The fact that we found that ATRX is overexpressed in a wide variety of human cancers prompted us to study in the *Drosophila* model what would be the molecular effects of overexpressing dADD1 proteins since this domain directs the protein to heterochromatin [25]. We used the UAS-GAL4 system to modify the levels of dADD1 protein isoforms. First, we directed the expression of all dADD1 protein isoforms using a ubiquitous driver (Actin or Tubulin), making genetic crosses between the flies that carried the Actin or Tubulin drivers as described in the Methods Section with the *UAS-dADD1* lines which resulted in organism lethality (Table 1). Since we had determined in our previous publication that the dAdd1a and dAdd1b protein isoforms have different activities, we overexpressed them individually to evaluate their contribution to organism lethality. We achieved similar results when we over-expressed either dADD1a or dADD1b protein isoforms (for a brief description of these lines please refer to the Methods section) [16] (Table 1), and in all the cases the organisms died at early stages of development (data not shown).

**Table 1:** Effect of dADD1 overexpression in organism viability

Genotype	Viability ^a^ (%)
*+/+;Tub-GAL4/+*	352/372 (94)
*+/ UAS-dADD1;+/+*	361/361 (100)
*+/+; UAS-dADD1a/+*	558/558 (100)
*+/+; UAS-dADD1b/+*	273/273(100)
*+/UAS-dADD1;Tub-GAL4/+*	0/361 (0)
*+/+;Tub-GAL4/UAS-dADD1a*	0/558 (0)
*+/+;Tub-GAL4/UAS-dADD1b*	0/273 (0)

^a^The number of flies gotten over the number of adult flies expected to agree with the healthiest class in each cross

Because the ubiquitous over-expression of all or two of dADD1 isoforms led to organism lethality, we decided to drive the expression of dADD1 directly to the salivary glands using the *Sgs3-GAL4* driver (as described in the Methods section) and evaluate chromosome architecture in polytene squashes. In wild-type salivary glands, the chromosome shows the characteristic pattern of bands and interbands in the chromosome, we can also see the chromocenter (dashed box in 1a and 1a’), the telomeres (continuous line boxes in 1a and 1a”) and the overall chromosome integrity (Fig. 1a). Overexpression of dADD1 results in a general loss of the chromatin structure (Fig. 1b), the chromocenter becomes fragile (compare dashed boxes of Fig. 1a‘and b’), there is a loss of the banding pattern in some regions at the chromosome arms, at the telomeres (arrows and continued line boxes in Fig. 1b and an amplified image in 1b”) and dissociated chromosome copies.

To confirm the overexpression of *dADD1* mRNAs, we analyzed the transcript levels in salivary glands obtained from the *UAS-dADD1;Sgs3-Gal4* genotype and compared them to the wild-type salivary glands using the primers described in [15] which are specific for every isoform, *Rp49* primers were used as control. We found that all of the isoforms mRNA levels were two to three-fold higher than the wild-type, compare lanes 1 and 3 in Fig. 1c, which is similar to the fold change observed in the tumors from the COSMIC database (data no shown). Additionally, we used the previously described pan-dADD1 antibody to perform a western blot from total protein extract of salivary glands [15] and found that dADD1 levels were overexpressed at least three-fold in comparison to the wild-type protein levels (compare lanes 1 and 3 in Fig. 1d).

We evaluated changes in DNA and histones content in both wild-type and overexpressed conditions to rule out differences in endoreduplication cycles and chromatin content and we did not observe any significative changes between the wild-type and dADD1 overexpression conditions (See Additional file 2a and b).

The observed phenotype of an overall loss of banding patterns and chromosome compaction when we overexpress dADD1 was somewhat unexpected since, as mentioned above, we recently reported that dADD1 participates in the silencing and compaction of the telomeric retrotransposons and prevent telomeric fusions. Therefore, we expected that the overexpression could lead to an opposite localized effect (that is more compaction) in some areas of the chromosomes, but this was not the case. In our previous report, we performed rescue experiments in a null *dadd1* background and found that HP1a was restored to the telomeric region when the rescue was performed with dADD1a isoform. However, we also observed that upon dADD1a overexpression, HP1a was lost from the chromocenter in a dADD1a dose-dependent manner [16].

**Fig. 2:**
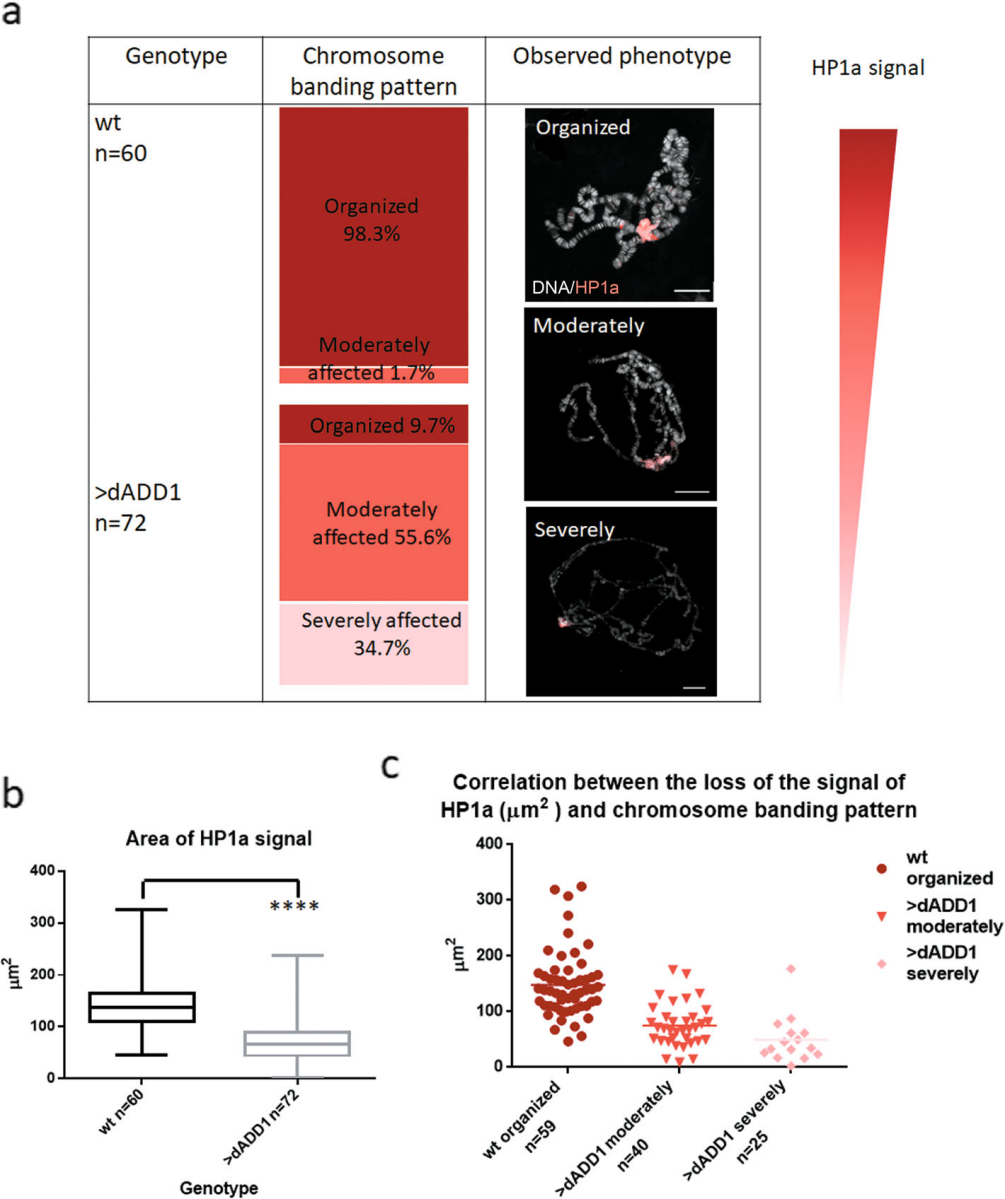
dADD1 overexpression induces HP1a delocalization, which correlates with loss of chromosome banding pattern at the chromocenter. **a** Classification of the severity of the phenotypes and the amount of HP1a signal observed in the polytene chromosomes. The first column indicates the genotypes analyzed; the second column shows the classification of the chromosomes according to their banding pattern in organized, moderately and severely affected (see text for a detailed explanation on the classification). Different shades of red indicate the intensity of HP1a signal. The third column shows an example of each of these polytene squashes where DNA is evidenced by DAPI staining (shown in gray), HP1a (red) scale bar 20 μm. **b** Area of HP1a signal quantified with ImageJ. The number of chromosomes analyzed for each condition is shown with “n,” an Unpaired t-test was performed to determine significance, the Area of HP1a signal is diminished in dADD1 overexpressing chromosomes (>dAdd1 vs. wt). **c** Correlation between HP1a and loss of chromosome banding pattern. Ordinary one-way ANOVA was performed to determine the significance, *p* < 0.0001****

To get further insights into the mechanisms involved in the emergence of these phenotypes, we performed HP1a immunolocalizations in wild-type and dADD1 overexpressing chromosomes. We performed all the immunolocalization experiments to get the overexpressing and wild-type chromosomes at the same time to avoid differences that could be introduced by the immunohistochemistry assay. To evaluate if there was a correlation between the loss of banding pattern and HP1a localization we quantified the number of chromosomes which presented a loss of banding pattern and divided the chromosomes into three groups according to the severity of banding pattern loss: organized (well-defined bands, chromocenter with HP1a signal), moderately affected (still with HP1a signal in the chromocenter but with irregularities in the banding pattern), and severely altered (very little or no signal of HP1a in the chromocenter, many defects in the pattern of banding and thickness of the chromosome arm), examples of each phenotype and classifications are shown in Fig. 2a. The wild-type chromosomes conserved the banding pattern and also the HP1a pericentromeric signal, and only 1.7% of the chromosomes had the chromosome banding pattern moderately affected upon the squashing technique; also, the vast majority (98.3%) had a strong HP1a signal. In dADD1 overexpressing chromosomes (>dADD1), only 9.7% of the chromosomes analyzed appeared to maintain an organized chromatin structure, but the majority (90.3%) did not (Fig. 2a). We quantified the area of HP1a signal at the chromocenter (see methods section) and was reduced in dADD1 overexpressing chromosomes (Fig. 2b). To evaluate if the chromosome banding pattern was affected due to HP1 loss, we measured the results obtained from each genotype and HP1a signal intensity, and we observed that indeed, chromosomes have banding pattern loss had less HP1a bound to the chromocenter (Fig. 2c). These experiments indicate that over-expression of dADD1 in a wild-type background results in loss of heterochromatin and compaction mediated at least in part by HP1a loss.

### HP1a is delocalized in dADD1 overexpressing cells

In the previous experiments, dADD1 overexpression results in HP1a loss from the chromocenter (Fig. 2). We performed HP1a immunofluorescences in the salivary glands to evaluate whether the loss of HP1a from the chromatin was a result of a change in its sub-nuclear localization.

**Fig. 3:**
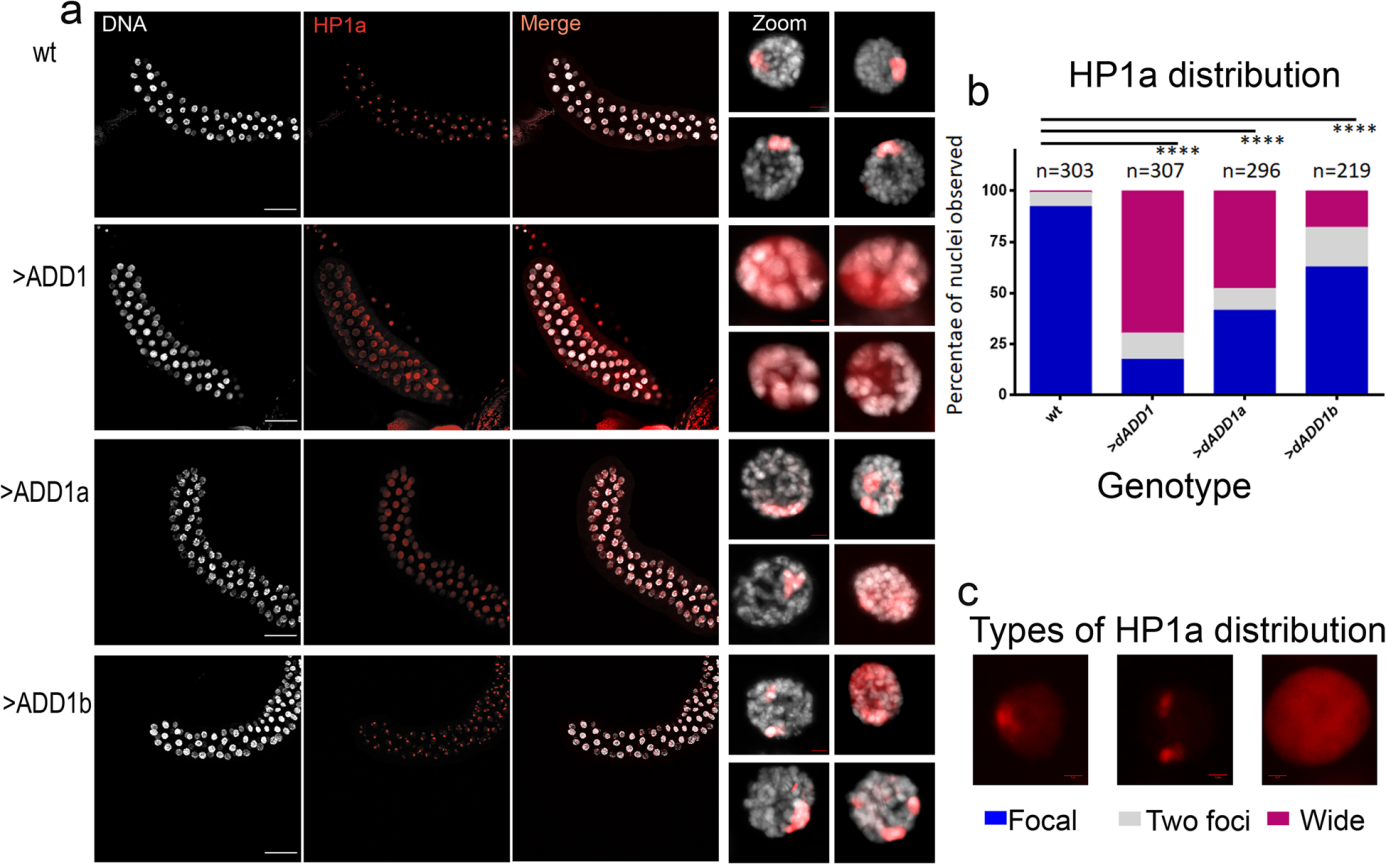
Overexpression of dADD1 proteins changes HP1a distribution in whole salivary glands. **a** Salivary gland immunostaining with HP1a antibody from wild-type (wt), *UAS-dADD1; Sgs3-GAL4 (>dAdd1)*, *Sgs3-GAL4/UAS-dADD1a (>dAdd1a)* and *Sgs3-GAL4/UAS-dADD1b (>dADD1b),* HP1a (red signal), DNA (grey signal) and merge scale bar 100 μm. The right column shows a magnified image of a single nucleus with scale bar 5 μm **b** Percentage of nuclei with three different distributions of HP1a signal, “n” represents the number of nuclei analyzed in each genotype. Kruskal-Wallis Test was performed to determine the significance, *p* < 0.0001****, indicating a scale “1” for focal, “2” for two foci and “4” for wide. **c** Classification of HP1a (red signal) distributions found in the nuclei, scale bar 5 μm

We directed the expression of dADD1a and dADD1b or all isoforms using the *Sgs3-GAL4* driver to salivary glands and performed HP1a immunofluorescences (see additional File 2c for specific mRNA expression of each isoform evaluated by RT-PCR). In wild-type salivary glands, HP1a appears as a single focalized spot that marks the chromocenter [26]. Figure 3a, first row, and magnified images. Almost 92% of the nuclei counted presented this phenotype; the rest (7%) showed two foci (Fig. 3b and c). Overexpression of dADD1 results in the loss of the HP1a focalized signal, 17% of the observed nuclei presented a wild-type focal distribution, whereas 70% showed a wide distribution in which the HP1a signal appears to be distributed in the nucleoplasm with not bright enriched foci, see also the magnified images of the nuclei (Fig. 3a, second-row genotype *> dADD1*, Fig. 3b second column) and 13% presented the two foci phenotype.

According to these results, we can envision that the HP1a protein present in the nucleoplasm is most likely lost during the squashing technique; this can account for the lack of HP1a protein in the polytene squashes. On the other hand, the expression of either isoform results in a mixture of the possible phenotypes shown in Fig. 3a, third and fourth row, and magnified images.

In dADD1a overexpression, 48% of the chromosomes presented a wide HP1a distribution (Fig. 3b third column marked >dADD1a). In contrast, dADD1b overexpressing cells exhibited close to 18% wide distribution and had the highest percentage of two foci distribution (19%), but the majority had a wild-type (focal) HP1a distribution (63%) (Fig. 3a, fourth row, and Fig. 3b, 4th column). Overexpression of all dADD1 isoforms had the most profound effect in disrupting the HP1a focal signal, with almost 70% of the observed chromosomes presenting a wide HP1a distribution. Looking at the percentages, the combined dADD1a and dADD1b overexpression is responsible for nearly all of the 70% of HP1a wide distribution (48% plus 18% = 66%) (Fig. 3b), however dADD1a has the most marked effect on HP1a distribution, this could be a result of the reported interaction between these two proteins [14, 27].

We also quantified the area of the nuclei to see if it changed upon dADD1 overexpression, but we could not find any significative differences when we compared it to the control nuclei (Additional file 2d), next we measured the intensity of the DAPI, and we did not observe any differences. These data suggest that dADD1 overexpression does not affect the size of the nuclei nor the amount of DNA in polytene cells (See Additional file 2 d and e).

As mentioned before, dADD1 protein isoforms conserve a common region, which includes the ADD domain, the difference between them is the presence in the carboxy-terminal end of additional MADF domains in the dADD1b protein isoform which are not present in the dADD1a isoform (Fig. 1e). Therefore, the common region may have an essential role in disrupting HP1a foci. The ADD domain recognizes and binds to H3K9me3 in combination with H3K4 without any modification; thus, it was possible that dADD1 overexpression could “compete” with HP1a for binding to the H3K9me3.

### H3K9me3 chromatin signal is lost upon dADD1 overexpression

We anticipated at least two possible scenarios. In the first one, dADD1 proteins could “deplete/remove” HP1a from the chromocenter of salivary glands directly via protein-protein interactions. The second one could involve a competition between HP1a and dADD1 for the H3K9me3 binding site [16].

It has been widely demonstrated that H3K9me3 post-translational modification is needed to maintain pericentric heterochromatin and that this histone mark is enriched at the chromocenter of polytene chromosomes [28]. Any perturbation of this histone mark leads to HP1a loss from the chromocenter [29, 30]. Therefore, we decided to evaluate if this mark was conserved in dADD1 overexpressing cells.

**Fig. 4:**
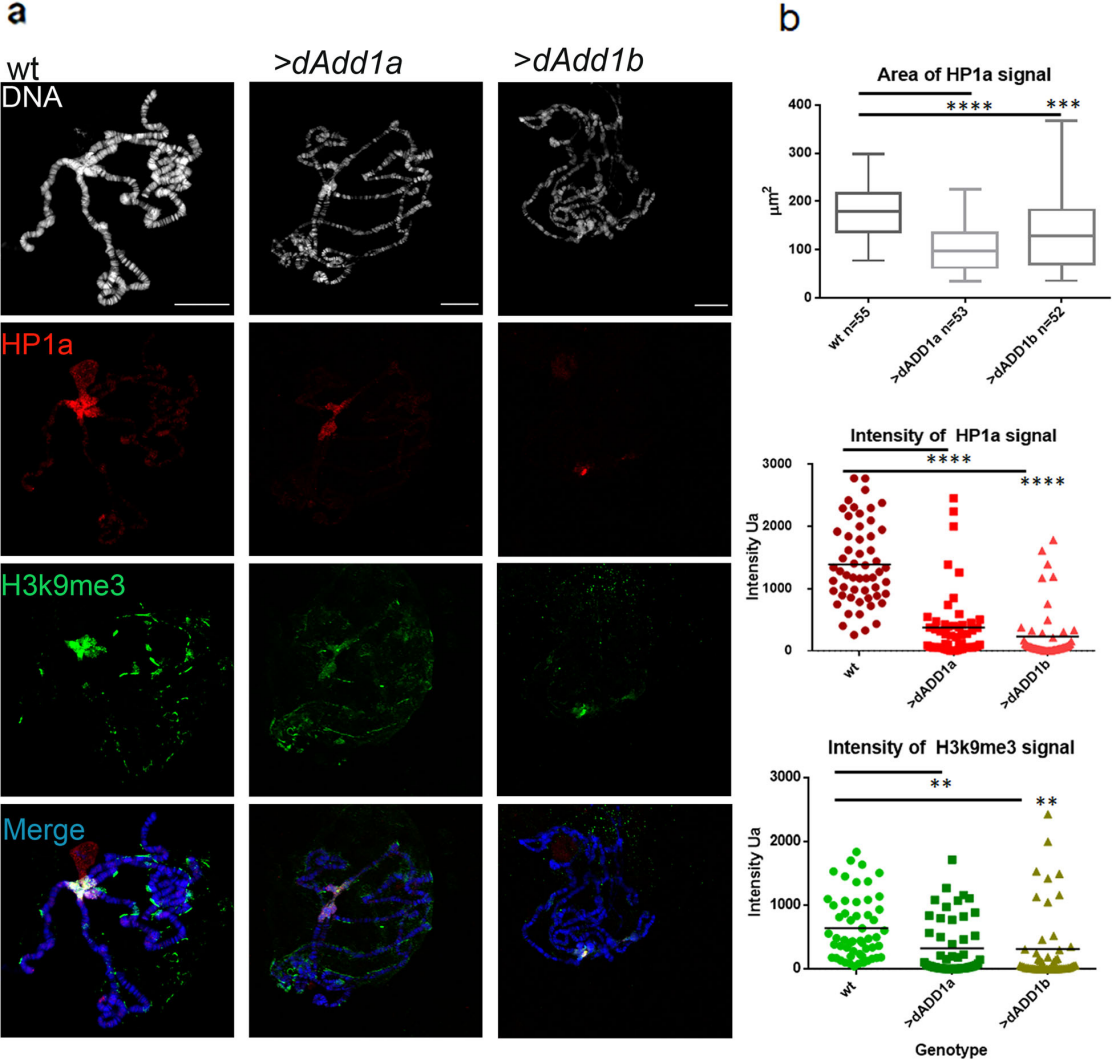
Overexpression of dADD1 proteins disturbs H3K9me3 signal. **a** Immunostaining of polytene chromosomes from wild-type, S*gs3-GAL4/UAS-dADD1a (>dAdd1a)* and S*gs3-GAL4/UAS-dADD1b (>dADD1b),* DNA (DAPI staining shown in grey), HP1a (red signal), H3K9me3 (green signal) and merge scale bar 20 μm. **b** The signal area of ​​HP1a was quantified by ImageJ as well as the intensity of HP1a and H3K9me3 signal. The “n” represents the number of chromosomes analyzed in each genotype; an Ordinary one-way ANOVA test was performed to determine significance *p* < 0.05*, < 0.01 **, < 0.001***, < 0.0001****

We performed double immunostaining of H3K9me3 and HP1a in polytene chromosomes from wild-type or overexpressing dADD1 cells. Representative confocal images are shown in Fig. 4. In wild-type chromosomes, the H3K9me3 (green) and HP1a (red) signals are enriched at the chromocenter as previously described [28], all of the wild-type chromosomes analyzed presented both signals (100%) (Fig. 4a, first column, and Additional file 3a first row). To explain the differences between the wild-type and the overexpressing chromosomes, we quantified the area of HP1a signal on the chromosomes and the intensity of HP1a and H3K9me3 (as described in the methods section). The area of the signal helped us determined the “spreading” of the HP1a domain, and the intensity is a direct measure of the chromatin-bound HP1a or H3K9me3 histone mark. When dADD1 is overexpressed, there was less HP1a signal, and also H3K9me3 was affected (see Additional file 3a third row). When either dADD1a or b protein isoforms are overexpressed, the chromatin banding pattern is still maintained, but there is less HP1a signal (Fig. 4a middle and right column and Fig. 4b), the HP1a quantified area is slightly less affected by the dADD1b overexpression. However, this effect could be because the majority of the chromosomes had a “split” HP1a signal (a representative chromosome is shown in Fig. 4a, right column, and Fig. 4b “area” plot) as if the chromocenter is more fragile and prone to “break” upon the squashing treatment. The signal intensity of both H3K9me3 and HP1a is diminished in the overexpression of both isoforms (Fig. 4b, intensity plots).

Overexpression of dADD1a or dADD1b also affects their own chromosome localization. In dADD1a overexpression, the signal at the chromocenter is lost, and the banding pattern looks more defined than the wild-type, whereas, in the case of dADD1b overexpression, the dADD1 pattern looks punctuated instead of bands but also the signal at the chromocenter is reduced (Additional file 3b).

These results indicate that dADD1 overexpression is not competing with HP1a to bind to its chromatin recognition site, but it could somewhat affect the chromocenter by titrating HP1a or HP1a containing complexes.

### dADD1 misexpression affects the expression of some heterochromatic genes

Some genes embedded in heterochromatin need this surrounding context for their correct expression and also require the presence of HP1a [31]. Since HP1a delocalized from many regions upon dADD1 overexpression, we decided to analyze if the expression of these genes was also affected.

We used as a selection guide DamIP chromatin profiling technique in Kc cells [32]. We have certainty of well-known genes located in pericentromeric regions that need HP1a for their proper expression in salivary glands such as *cinnamon (cin)*, *CG7742* [33], *light* (*lt)* [34], and *concertina (cta)* [35] these loci are in different chromosomes and heterochromatic regions [32, 35]. Of others, we decided to evaluate them because they are silenced in salivary glands, such as *kraken (Kra)* and *P-element somatic inhibitor (Psi)* [36]. We also evaluated euchromatic genes as control regions which are not controlled by either HP1a or Su(var)3–9. Using quantitative RT-PCR (qRT-PCR), we analyzed the transcript levels of all these genes in wild-type, dADD1a or b overexpressing salivary glands and also in the null *dadd1* organisms because in our first dADD1 report, we demonstrated the co-localization of HP1a and dADD1 proteins in polytene chromosomes, particularly at the chromocenter, but also at some bands throughout the polytene arms and chromosome four [15].

**Fig. 5:**
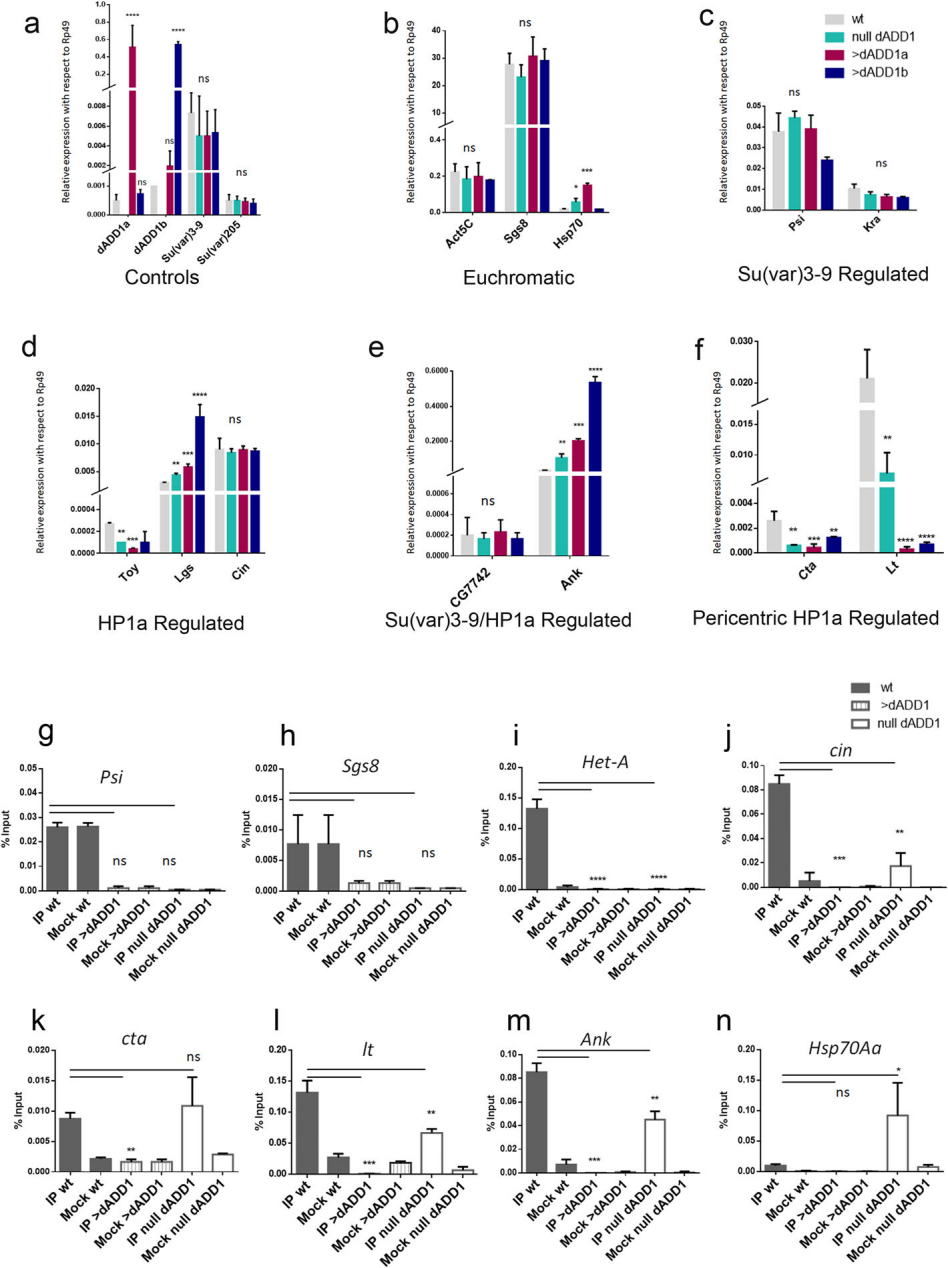
dADD1 overexpression results in transcriptional silencing of some genes and loss of HP1a. qRT-PCR analyses, wild-type (gray bars), null dADD1 (aqua bars), *Sgs3-GAL4/UAS-dADD1a* (red bars) and S*gs3-GAL4/UAS-dADD1b* (dark blue bars). Three independent biological replicates were performed. Data are shown as expression relative to *Rp49* transcript. Ordinary one-way ANOVA was executed to determine significance with *P*-values (*p* < 0.05*, < 0.01 **, < 0.001***, < 0.0001****, ns, no significance). **a** controls, **b** euchromatin genes, **c** genes regulated by Su(var)3–9, **d** genes regulated by HP1a, **e** genes regulated by Su(var)3–9 and HP1a, **f** pericentric genes. HP1a protein enrichment at different analyzed promoters. Chromatin immunoprecipitation experiments were performed using the C1A9 antibody (anti-HP1a) and mouse IgG as Mock. *Psi*, *Sgs8* and *Hsp70* are euchromatic regions without HP1a (**g** and **h** and **n**); note there is no enrichment versus the mock in the wild-type condition. Promoters of the Het-A retrotransposon (**i**) and *Cin* (**j**) were evaluated as telomeric and sub-telomeric regions in which HP1a is localized. Cta (**k**) and Lt (**l**) are pericentromeric, and HP1a also regulates them. *Ank* is regulated by Su (var)3–9 and HP1a (**m**). Error bars represent standard deviation. *P*-values (*p* < 0.05*, < 0.01 **, < 0.001***, < 0.0001****, ns, no significance)

We analyzed public data in which they used a Bio-tap tagged dADD1a for ChIP-seq experiments in S2 cells and evaluated the presence of dADD1a in the same genes of Fig. 5. Although S2 cells are embryonic, the position of the genes in the chromosome does not change, and neither does constitutive heterochromatin; therefore, we believe the ChIP-seq data helped us to get further information on dADD1a position in other heterochromatic gene domains. We found that dADD1a is not present in euchromatic genes such as *Sgs8* and A*ctin*, neither in *Su (var)205* and *Su (var)3–9* nor in genes controlled by Su(var)3–9 such as *Psi* and *kraken* see Additional file 5.

When we looked at genes controlled exclusively by HP1a [37], we only found dADD1a at *toy* and *lgs,* which are located at chromosome four, which is mainly heterochromatic, whereas in *cin* there is not dADD1a (Additional file 5).

In HP1a/Su (var)3–9 controlled genes, dADD1 is localized in *Ank* but not in CG7742. There is also the presence of dADD1 in the pericentromeric genes, *lt* and *cta* (Additional file 5). dADD1a is located at the promoter, but also through the gene bodies of the analyzed genes.

For the qRT-PCR, we used *Rp49* transcript for data normalization since the transcript levels remained very similar in the wild-type and the *dadd1* null or *dadd1* overexpressing salivary glands (data not shown). All the transcripts were analyzed in four genetic backgrounds, wild-type (gray bars), null *dadd1* (aqua bars), *dadd1a* overexpression (red bars) and *dadd1b* overexpression (dark blue bars) (see Fig. 5). First, we analyzed the transcript levels of *dADD1a or b* (Fig. 5a top panel labeled as “controls”). When we overexpress each isoform, higher levels of *dADD1a* or *dADD1b* transcripts were obtained when compared to the wt as expected (Fig. 5a). Next, we analyzed if heterochromatin maintenance genes such as *Su (var)3–9* or *Su (var)205* (the gene that encodes HP1a) transcript levels were affected, but the transcripts remained at similar levels (Fig. 5a), suggesting that the previously observed effects on loss of HP1a and H3K9me3 (Figs. 3 and 4) are not due to loss of transcription of these genes.

Neither euchromatic genes such as *Act5C* and *Sgs8,* nor Su(var)3–9-regulated genes were affected (Fig. 5b, c). The only affected genes were HP1a-regulated genes such as *Toy* and *Lgs* (Fig. 5d); Su(var)3–9/HP1a regulated genes such as *Ank* and pericentric genes such as *Cta* and *light* (Fig. 5f). The majority of these genes were down-regulated in the null *dadd1* organisms or in the overexpression of either *dadd1a* or *b* isoforms. It is worth mentioning that all of these genes also are bound by dADD1 in S2 cells. The only two genes that showed an over-expression either in the null *dadd1* or *dadd1* overexpression genetic backgrounds were *Lgs* and *Ank*, which were overexpressed in all genetic backgrounds when compared to the wild-type. Another gene that resulted de-regulated was *hsp70*; in both the null *dadd1* organisms and *dadd1a* overexpression the transcript was more abundant.

The scheme in Additional file 4 shows the location of the analyzed genes. Particularly, *Lgs* and *Ank* are located within chromosome 4, which is highly heterochromatic but are not pericentric or telomeric. Therefore lack of dADD1 proteins or their overexpression, results in different outcomes depending on the analyzed gene and their chromosomal localization. Down-regulation of heterochromatin embedded genes could be explained by the loss of HP1a in the chromatin, which is known to be required for the correct transcription of some of these genes [37, 38]. To address if there were changes in HP1a binding to these genes, we performed chromatin immunoprecipitation followed by quantitative PCR (ChIP-qPCR). First, we analyzed two genes which are not regulated by HP1a, *Psi* and *Sgs8* do not show any HP1a enrichment versus the mock condition (IgG antibody, see Methods Section and Figs. 5 g and h), next we analyzed a region which we know is target of HP1a, the *Het-A* promoter, as we can see in Fig. 5i, there is an enrichment of HP1a at this region in wild-type individuals. In *dadd1* null organisms, HP1a is no longer present at this site, coinciding with our previous publication; importantly, overexpression of dADD1 proteins also leads to loss of HP1a at this telomeric region. The same effect can be seen in the *light*, *Ank* and *cin* regions, in the case of *light* and *Ank*, we can see that the loss of HP1a leads to opposite effects in gene transcription, *light* is down-regulated and *Ank* is up-regulated, this indicates that there is a differential role for HP1a at these heterochromatic sites, surprisingly, although *cin* loses HP1a in both the null *dadd1* and *dadd1* overexpression, the transcript is not affected (Fig. 5d and j). *cta,* which is also a pericentromeric gene and was down-regulated in all the conditions analyzed, conserves HP1a in the null *dadd1* organisms (Fig. 5k). Another unexpected result came when we analyzed *Hsp70Aa* expression; in null *dadd1* organisms and dADD1a overexpression conditions, this gene is up-regulated, and in a wild-type background, HP1a is not present at this gene, however, in salivary glands from organisms that lack *dadd1*, HP1a becomes enriched at this promoter.

These results demonstrate that dADD1 proteins are important to achieve correct levels of expression of some genes embedded in sub-telomeric or pericentromeric heterochromatin, genes located in chromosome four, which is mainly heterochromatic, but also, at some euchromatic loci.

Overall, our data indicate that misexpression of dADD1 (either over or under expression) has an important impact on chromatin structure and in the localization of HP1a proteins, which leads to differences in heterochromatic and euchromatic gene expression.

### dXNP localization is altered upon dADD1 misexpression

The differences observed in gene expression are not only due to HP1a loss, so dADD1 proteins may cooperate with other protein complexes or proteins to control gene expression.

**Fig. 6:**
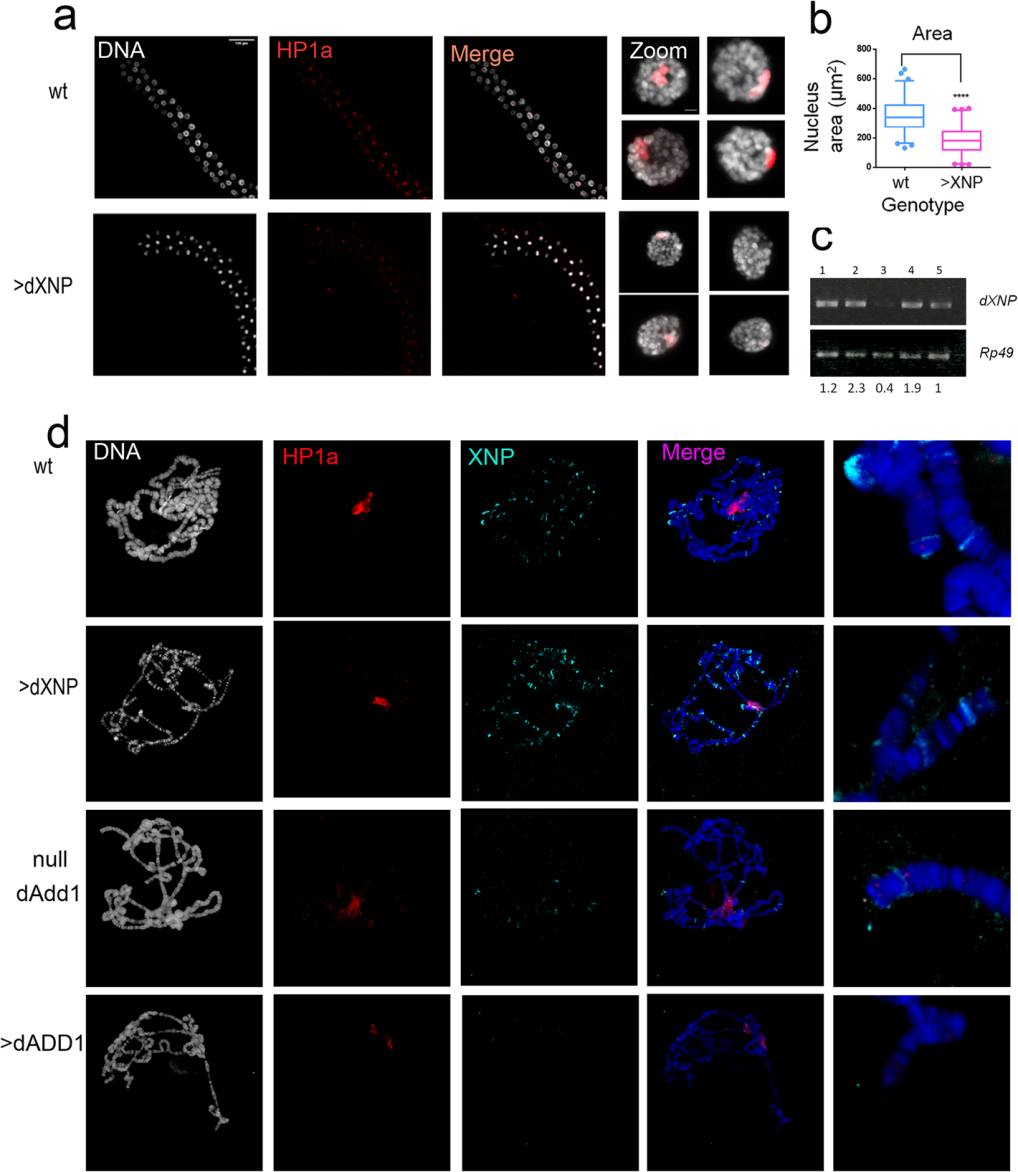
dADD1 overexpression causes changes in dXNP binding. **a** Salivary gland immunostaining with HP1a antibody from wild-type (wt) and dXNP overexpression *(>dXNP) Sgs3-GAL4/UAS-dXNP*. HP1a (red signal), DNA (grey signal) and merge (scale bar 100 μm). In the right column a magnification of a single nucleus is shown (scale bar 5 μm). **b** Nucleus area quantification showing a reduction in *(>XNP),* wt *n* = 341 and XNP *n* = 344. **c** RT-PCR analysis of the transcript levels of dXNP in salivary glands. Lanes in the gel correspond to 1) wild-type (wt) 2) *Sgs3-Gal4*/*UAS-XNP (>XNP)*, 3) *xnp*^*2*^*/xnp*^*3*^ (a heteroallelic condition in which *dxnp* transcripts are diminished), 4) *dadd1*^*2*^*/ dadd1*^*2*^ (null *dadd1*) and 5) *UAS-dadd1*; *Sgs3-GAL4*(>dADD1), genotypes. *Rp49* transcript was used as a control; the numbers below are the bands quantification with respect to *Rp49* signal intensity. **d** Immunostaining of polytene chromosomes from wild-type (wt), *Sgs3-GAL4/UAS-XNP* (>XNP), *dadd1*^*2*^*/ dadd1*^*2*^ (null dAdd1) and *UAS-dADD1; Sgs3-GAL4* (>dADD1), DNA (DAPI staining shown in grey), HP1a (red signal), dXNP (cyan signal) and merge. The right column presents a magnification of the telomeric regions

Previous work from our lab identified dXNP as a dADD1 interactor [15]. We decided to analyze if overexpression of dXNP phenocopied the loss of heterochromatin observed upon dADD1 overexpression. We performed genetic crosses between *Sgs3-Gal4* and *UAS-dXNP* lines to direct the expression only to salivary glands. First, we looked at HP1a distribution in complete salivary glands. We found that HP1a focal distribution is maintained, however, when we compare dXNP overexpression to wild-type salivary glands, the chromatin seems more compacted, and the HP1a signal although present, is diminished, see Fig. 6a. We measured the nuclei area and found that it is diminished upon dXNP overexpression (Fig. 6b). Then, we looked at polytene chromosome spreads and analyzed HP1a and dXNP distribution. In wt chromosomes, these proteins co-localized and we also saw telomeric localization of dXNP, as expected, Fig. 6d first row and magnified images. Upon dXNP overexpression, the number of bands increased, as well as the signal at certain heterochromatic regions such as the telomeres (Fig. 6d second row), however, in null *dadd1* organisms or dADD1 overexpression, the dXNP signal diminishes, and only a few numbers of bands conserve dXNP protein signal, see Fig. 6d last two rows. Then we analyzed the *dXNP* transcript levels and are normally expressed (compare lines 1 versus 4 and 5 in Fig. 6c). Therefore, lack or overexpression of dADD1 does not affect transcription of this gene, only its chromatin location.

These results demonstrate that upon dADD1 loss, dXNP loses its wild-type chromatin localization as fewer dXNP bands are detected in these chromosomes, which could lead to loss of chromatin compaction. Interestingly, upon dXNP overexpression, there seems to be general chromatin compaction that coincides with the presence of more dXNP bands at polytene chromosomes. These results support the role of dADD1 as essential proteins to maintain a correct chromatin organization, protein localization, and gene expression.

## Discussion

Maintenance of a correct chromatin structure is central for cell viability. During the transformation process in cancerous cells, many genes become deregulated, changing several protein levels and allowing the cell to escape normal controls of cell cycle and gene regulation. Currently, many association studies address the role of loss of function mutations of many transcriptional factors and chromatin remodelers in cancer cells, and many databases have been able to concentrate these data to understand this important disease.

ATRX loss of function mutations has been associated with several different cancer cells, from glioblastoma to pancreatic cancer [21]. The number of these mutations has grown in the past few years, and many of them affect the domains important for the wild-type function of the protein [39]. Although the majority of the studies have focused on the loss of function mutations, many reports show overexpression of ATRX in different types of cancer. During the transformation process of the cells, many genes become deregulated and overexpressed, and there is a need for a simplified model to address the roles of the overexpression of these genes.

In this work, we report that somatic cancer cases that have ATRX overexpression are more represented in the databases examined than the under-expression conditions. This data was unexpected since most studies in the functions of ATRX in development come from the loss of function mutants. However, there have also been a few studies in which this protein has been overexpressed, leading to the appearance of similar phenotypes observed in loss of function mutants [13].

Our investigations on the function of dADD1 have led us to develop tools that help us address several questions on the purpose of these proteins. Additionally, the domains are separated in *Drosophila,* and we can study the independent roles of the ADD and the SNF2 domains of the ATRX orthologue.

In this work, we analyzed the effects of the misexpression of one of the central ATRX domains, the ADD domain. This domain shares more than 52% homology with the ADD domain of the human ATRX and also recognizes the combination of K9me3 and unmethylated K4 of histone H3 [14]. The *dadd1* gene encodes three different protein isoforms which conserve the ADD domain; our group has demonstrated that dADD1 proteins are involved in the maintenance of chromosome stability and heterochromatin by tethering HP1a to the telomeres. Additionally, the isoforms participate in the silencing of the telomeric retrotransposons, which in somatic cells are repressed and also direct HP1a binding to the telomeres [16]. Deregulation of this silencing and the lack of HP1a at the telomeres leads to the appearance of chromosomal aberrations and genome instability.

Overexpression of dADD1 proteins resulted in chromatin structure loss, and it caused a general decompaction and the dissociation of chromatin fibers around the chromocenter (Figs. 1 and 2). These phenotypes were stronger when overexpression of all dADD1 isoforms was performed. In wild-type salivary glands, the transcript levels of *dADD1* are low compared to other analyzed transcripts (see Fig. 5). These results indicate that the levels of dADD1 in polytene cells need to be maintained on the lower side to achieve a correct chromatin structure. Recently, Mitzi Kuroda’s group identified dADD1 as a strong HP1a interactor and also other factors such as the methyltransferases Eggless/dSETDB1 and Su (var)3–9. These methyltransferases participate in the formation and maintenance of pericentric heterochromatin [39, 40]. Loss of either of these methyltransferases or a shift in their levels affects pericentric heterochromatin H3K9 methylation and HP1a localization [39, 40]. Given that dADD1 interacts with these histone methyltransferases, one possibility is that overexpression of dADD1 proteins breaks the stoichiometry of complexes containing these proteins, affecting the methyltransferases activity, leading to loss of the methylation mark, and HP1a (see Figs. 2 and 4). It is also fundamental to consider that the maintenance and propagation of H3K9me3 also require HP1a since it recruits Su (var)3–9; therefore, disruption of HP1a from the chromocenter might lead to the observed changes in H3K9me3.

Reduction in H3K9me3 signal and HP1a from the chromocenter partially explains the loss of global chromatin structure observed in the polytene chromosomes. Our group has demonstrated that dADD1 also interacts physically with the ATPase dXNP; this protein does not have a DNA or chromatin binding domain. Consequently, it relies on the interaction with other proteins to reach its targets on the chromatin [41]. dXNP is important for the maintenance of beta heterochromatin and has been shown to interact also with HP1a [42, 43]. dADD1 levels can change HP1a localization, and also affect dXNP binding to chromatin leading to a deregulation of the ATPase activity and loss of chromatin structure, see Fig. 6 [43].

Also, overexpression of either isoform (Additional file 3b) affected dADD1 chromatin binding; therefore, the model of a complex between HP1a, dADD1, dXNP, and a methyltransferase which maintain not only telomeric but pericentric heterochromatin is further supported by our data.

Observations have been made in which overexpressing dXNP in the developing eye and wing causes apoptosis through the JNK pathway [4, 44]. In other studies, over-activation of JAK phosphorylates STAT and STAT92E phosphorylation results in chromatin disruption and loss of HP1a stability [45]. Otherwise, reducing levels of phosphorylated STAT92E or its loss also causes instability in heterochromatin [46]. Both JNK and JAK-STAT are two of the signals that play a primary role during cell fate [47]. This data could be noteworthy for our work because the over-expression of dADD1 proteins could lead to apoptosis via JNK, and it is well-known that STAT abnormal activation by phosphorylation is related to human cancers [48]. Further studies will be needed to clarify this point.

In the literature, there are reports of mutants that phenocopy the chromocenter loss of organization that we observed when we overexpress dADD1. Mutants affecting H3.3 levels or a mutant that substitutes the lysine 9 for an arginine lead to a disorganization of the chromocenter [48, 49].

Vertebrate ATRX is capable of interacting with the histone chaperone DAXX and exchange the H3.3 variant at different heterochromatic regions such as the telomeres and pericentric heterochromatin [25] also, the H3.3 that is deposited helps to maintain the levels H3K9me3 necessary for proper heterochromatin maintenance [25, 50]. In *Drosophila,* the DAXX like protein has been shown to cooperate with ASF1 for the deposition of H3.3 and also with dXNP at certain heterochromatic regions [51]. Thus, it is possible that also dADD1 proteins may be cooperating with this complex to maintain heterochromatin, however to date, there are no studies on histone variant H3.3 in a null *dadd1* background or overexpression condition, but it would be a critical and interest aspect for future research to thoroughly understand the cooperative roles of these proteins.

Heterochromatic foci are needed to maintain a correct chromatin conformation. Proteins that can disrupt these foci may have significant roles as drivers of disease [52, 53]. In our results, we can see that both tested dADD1 isoforms disrupt HP1a foci to different extents; however, the effect is more pronounced when we overexpress the “a” isoform, which has been directly co-precipitated with HP1a. The “b” isoform has additional MADF domains which could in part contribute to other of the observed phenotypes, it is known that in other proteins, the MADF domains recognize repetitive rich sequences which are also present in heterochromatic regions; still, more experiments are required to elucidate the MADF domains function.

It has been demonstrated that human HP1alpha drives phase separation in heterochromatin [54] a feature that is conserved in the *Drosophila* orthologue [54, 55]. Our results place dADD1 proteins as regulators of this HP1a property, probably maintaining a correct local concentration of HP1a oligomers at certain regions such as the telomeres and pericentric heterochromatin. Over and underexpression of dADD1 can disturb the concentration of HP1a and affect phase transition, which could lead to chromatin instability and alterations in gene expression [54, 55].

Our data demonstrate that dADD1 misexpression in the salivary glands affects HP1a, Su (var)3–9, dXNP and dADD1 localization, a set of genes show an important transcriptional effect, whereas other genes remain unaffected. Pericentric genes transcription was similarly affected upon dADD1 overexpression as in the null *dadd1* organisms (Fig. 5) and HP1a binding was also affected in both genetic backgrounds (see Fig. 5k and l) therefore at these pericentromeric genes dADD1 cooperates with HP1a to maintain a correct expression. Transcription from euchromatic genes such as *Sgs8* and *Act5c* was not affected; however the *Hsp70Aa* gene was upregulated in the null and dADD1a overexpression conditions and this effect seems to be independent on the presence of HP1a, this places dADD1 proteins as regulators of this chaperone which has also been observed to be over-expressed in different types of cancers [56]. Another interesting feature of this particular gene is that it has a “poised” Pol II, so it is possible that dADD1 misexpression could also be involved in controlling this “poised” state. It would be important to address if this or other heat-shock proteins which maintain correct homeostasis are also de-regulated in the human cancers in which ATRX is overexpressed.

In *Drosophila* salivary gland cells, gene transcription remains highly regulated despite the loss of HP1a foci and a concomitant loss of chromatin architecture. Chromatin from salivary glands is polytenized; thus, the transcriptional defects may be somehow “buffered” by other gene copies. This buffering effect may not be conserved in other cells and tissues as overexpression of dADD1 using a ubiquitous driver results in organism lethality (Table 1).

Contrary to what we observed with dADD1 overexpression, dXNP overexpression leads to chromatin compaction, evidenced by an increase of dXNP bands in polytene chromosomes, even at the telomeric regions and a decrease in the nucleus area (Fig. 6a and b). Also, it was highly difficult to obtain full spread chromosomes due to the high compaction (Fig. 6c). The dXNP protein lacks a DNA or chromatin binding domain, our group and others have shown that dXNP is able to interact with transcription factors such as DREF or chromatin-binding proteins such as HP1a. dXNP may reach chromatin via DREF or other factors and through the SNF2 domain promotes heterochromatinization, so an excess of dXNP could also affect gene expression or, as has been demonstrated, lead to cell death [45].

In the fly, this is the first study that presents the effect mediated by overexpression of the orthologues of the ADD and SNF2 domain of ATRX. Further investigation will be necessary and exciting to address the impact of dADD1 overexpression in a context in which we could suppress lethality, to evaluate if the cells acquire characteristics or phenotypes associated with cancer features. Also, we believe that further studies to understand why ATRX is overexpressed in the tumor cells is necessary. At present we know that this overexpression is not highly correlated to gene copy number loss or duplication which leads us to think that perhaps de-regulation of proteins that act in the control region of ATRX could be responsible for its overexpression, therefore, it would be interesting to understand the contribution of these factors to the transformed phenotype [57].

## Conclusions

The results presented here provide new evidence that dADD1 overexpression disrupts chromatin structure, affecting the localization of chromatin binding proteins such as HP1a, dXNP and H3K9me3 inducing chromosomal instability and organism death. Also, our group recently described dADD1 as a negative regulator of the expression of telomeric retrotransposons; however, in this study, we demonstrate dADD1 proteins are also required for correct heterochromatic and euchromatic gene transcription. Further genetic and biochemical characterization of dADD1 isoforms is necessary to understand their roles in the maintenance of chromatin stability and heterochromatic gene regulation.

## Methods

### Search on public data bases

Clinical relevance of mutation on ATRX was revised on the NCBI Genomic DATA Commons (GDC, https://portal.gdc.cancer.gov/) [58]. GDC is a general collecting portal that includes all cancer genomics studies data that the users are updating. Transcriptome expression pattern of ATRX on normal and cancer human samples were explored on the Human Protein Atlas (THPA, https://www.proteinatlas.org/, [58, 59] specialized proteome database and the manually curated Catalogue of Somatic Mutation in Cancer (COSMIC, https://cancer.sanger.ac.uk/cosmic, [41] last accessed at 18 February of 2019. Advanced search on COSMIC was filtered using the criteria of “Gene Expression (mRNA)” “over/under-expressed.” We downloaded each case, verified the full report and corroborated common samples between the Human Protein Atlas (THPA) and COSMIC databases. We selected the cases that do no report previous history of treatment (to avoid differences in expression due to chemical or irradiation treatments) and that had full available expression data.

### Fly stocks and genetic crosses

The wild-type flies used in this study were *w*^*1118*^. Fly stocks were maintained at 25 °C with standard food. All stocks were outcrossed with *w*^*1118*^; *Sp/CyO; TM6B, Tb*^*1*^*/MKRS* flies. *UAS-dADD1* (ID 200280 Kyoto stock center) were crossed to GAL4 drivers *Tub-GAL4* (ID 5138), *Act5C-GAL4* (ID 4414), *Sgs3-GAL4* (ID 6870). *His2Av-RFP* (ID BL23651), *UAS-XNP* (ID BL 26645), Xnp^2^ (ID BL 26643) and Xnp^3^ (ID BL 26644) were obtained from the Bloomington Drosophila Stock Center NIH P40OD018537. At least 100 flies were examined for each genotype. The generation of transgenic lines over-expressing of each isoform is explained in [16] Briefly, to generate the transgenic lines to conditionally direct the expression of dADD1 isoforms, the cDNA encoding either the A or B isoforms, was cloned into the pUAST vector carrying four UAS sequences [59]. Plasmid DNAs were sent to the Bestgene Company to obtain the transgenic *UAS-dADD1a* or *UAS-dADD1b* lines. Lines harboring insertions into the third chromosome were saved and balanced. The *dadd1*^*2*^ null allele was generously provided by Dr. Mitzi Kuroda and has been described in [14].

Immunostaining of polytene chromosomes, salivary glands, and signal quantifications.

Immunostaining of polytene chromosomes was performed as described in [41] with a modification in the spreading procedure with Lacto-acetic acid solution [60]. All controls and tested genotypes were processed at the same time to avoid variations in the immunohistochemistry procedures. Anti-HP1a (C1A9 from DSHB) antibody was used at 1:1000, anti-pandADD1 [15] and Anti-Histone H3K9me3 were used 1:50 (Abcam 8898) and anti-dXNP was used 1:10. Salivary gland immunohistochemistry was performed as described in [61]. Secondary antibodies Alexa fluor 488 or 568 (Invitrogen) were used at 1:300 and 1:100. Images were taken on a confocal laser scanning microscope (Olympus FV1000) with a 60x and 20x objective at the Laboratorio Nacional de Microscopia Avanzada (LNMA, UNAM). Images were processed using ImageJ. For polytene chromosomes, the intensity of the signal of HP1a or H3K9me3 was measured with ImageJ only by selecting the chromocenter and taking into account only the red or green signal in combination with the DNA signal (in blue) to eliminate the possible background.

### Western blot

Third instar wandering larvae were rinsed in ice-cold PBS 1X; 10 pairs of salivary glands were dissected from each analyzed genotype. Samples were boiled in Laemmli buffer, and proteins were separated in an 8% acrylamide/bis-acrylamide denaturing gels. Detection of the proteins was then carried out as previously described [15]. Anti-β-Tubulin (E7 from DSHB) antibody was used at 1:3000, and anti-pandADD1 was used as described before [15].

### Real-time RT-PCR assay

RNA was obtained using the Trizol reagent (Invitrogen) from 10 third instar larvae salivary glands. 1 mg of total RNA was converted to cDNA using reverse transcriptase enzyme and oligo-dT (Invitrogen). *Rp49* was used as a control for these experiments. The primers used for *dAdd1, a, b* and *Rp49* transcripts were the same as [15]. Other primer sequences are as follows (5′-3′):

**Table:** 

Name	Forward	Reverse
*Su (var)3–9*	GTGCGCTTCAAGAACGAACT	GCGGCCTTTTGGCAATTACT
*Su (var)205*	GGGCAAGAAAATCGACAACCC	GGCCATTATTGTCGGAGGCA
*Act5C*	GGTTGCAGCTTTAGTGGTCG	GGCACAGTATGGGAGACACC
*Sgs8*	TGCTCGTTGTCGCCGTC	GCCGCTCAAGACCCTCCATA
*Psi*	TCCAGGGAAAGAACGACGAA	CGCTCCAGATTGCTGGTTGA
*Kraken*	CGGAACTTTCGCCAGAGACAA	CTATCCGGCGAATCAGGCAT
*Toy*	CGTTGCGGAACGAACATCAT	CATCGTTGCAATCGGTTGTG
*Lgs*	GTACCACAACAGCAAACCCC	TGGGCTTGGTCGCCTACTTT
*Cin*	ACACGGTACAAAAGACCGCC	TCCACTTGCACTACGCAATCT
*CG7742*	ATGGCCAAGTGGAACGAACT	AATCCTCTGGCACTGAACCG
*Ank*	TTTCGTTCTTACGTGCTGCTC	TGTGCAAAGGGGTGAATCCT
*Cta*	ACGCGGCTTTGAGGAGTAC	GACTAGCTACCACAATATCC
*Lt*	TTTGAGGAGGCAATGGAACTT	CAGCCAGGCCGTCATAAAGA

Real-time PCR was performed as in [16]. Reactions were set up in duplicates, and the LightCycler Fast Start DNA Master SYBR Green 1 was used (Roche). Real-time quantitative PCR was performed by using a LightCycler 1.5 Instrument by Roche. PCR conditions were 95 °C for 10 min, followed by 40 cycles at 95 °C for 10s, alignment temperature for 10s, and 72 °C for 18 s. The alignment temperature was 65–60 °C. The threshold cycle (Ct) was used for assessing relative levels of respect to the housekeeping gene *Rp49*. The relative levels on mutant genotypes were compared to the corresponding levels on the wild-type strain to obtain the fold difference using the formula 2-ΔΔCT = [(CT gene of interest − CT internal control) sample A − (CT gene of interest − CT internal control) sample B] previously reported for relative transcript quantification [62]. Quantification of transcript abundance was measured with technical duplicates and three independent biological replicates were analyzed.

### Chromatin immunoprecipitation, qPCR, and data analyses

Chromatin immunoprecipitation from salivary glands was performed as [16] using Het-A primers. Data were expressed as % Input = 100*log2(dCt normalized ChIP), where dCt normalized ChIP = Ct sample − [Ct input*Input dilution factor)]. Two independent biological experiments were performed each with three technical replicates.

### Statistical analysis

All the graphs and statistical analyses were performed with GraphPad Prism 6. Data assuming normality and homogeneity of variance were analyzed with one-way ANOVA. Non-parametric data were evaluated with one-way ANOVA on ranks. Statistical significance was set at (*p* < 0.05*, < 0.01 **, < 0.001***, < 0.0001****). For data on the amount of DNA and Area of HP1a signal between two populations, an Unpaired t-test was performed. Kruskal-Wallis test was used to analyze data from HP1a distribution in salivary glands.

### Data analysis

Previously reported ChIP-seq data for S2 cells were obtained from Gene Expression Omnibus (GEO) database. GSM1363103, GSM1363104, GSM136105 and GSM136106 raw data from dADD1 (CG8290) [14]. Sequences were mapped to the dm6 reference genome using bowtie2 v 2.3.4.1 [63]. PCR duplicates were removed with samtools v1.7–2 [64]. Peak calling was conducted utilizing MACS v2.1.1.1 [65], requiring peaks to have a P-value of 1e-10. All genomics intersections were conducted with bedtools v.2.26 [66], peaks were called with the input control and visualized with the IGV genome browser [67].

## Supplementary information

**Additional file 1.** Distribution of cases by cancer type expressed as a percentage with a) ATRX overexpression and b) ATRX underexpression. Databases last accessed on February 18, 2019. C) Healthy breast sample tissue (Patient id: 3544) from a 45 years old female patient showing ATRX immunohistochemistry, d) Breast duct carcinoma sample (Patient id: 1874) from an 80-year-old patient. e) Breast duct carcinoma sample (Patient id: 4193) from a 43-year-old patient with a high signal of ATRX. f) Lobular carcinoma sample (Patient id: 4789) from a 49-year-old patient with a high signal of ATRX. g) Healthy Lung sample tissue (Patient id: 1678) from a 57-years-old female patient showing ATRX immunohistochemistry, h) Adenocarcinoma from female 51 years old (Patient id: 2041) showing low signal of ATRX, i) Squamous cell carcinoma from Male, 64 years old (Patient id:4090), j) Squamous cell carcinoma from Male, 72 years old (Patient id:4896) with high signal of ATRX. The immunohistochemistry was performed with the same antibody sc-15,408 from Santa Cruz Biotechnology. Image credit: Human Protein Atlas.

**Additional file 2 **The amount of DNA and chromatin does not change during dADD1 overexpression. a) DNA quantification in salivary glands over-expressing all the isoforms. An unpaired t-test was performed to determine significance. No significant differences were found. b) H2Av-RFP visualization of salivary glands with an H2Av-RFP transgenic line (red signal) the amount of chromatin between wild-type and over-expression of dADD1 does not change H2Av-RFP signal intensity*.* c) Transcript analyzes by RT-PCR lane 1) *Sgs3-GAL4*, lane 2) UAS-*dADD1a,* lane 3) UAS-*dADD1b,* lane 4) *Sgs3*-GAL4/UAS-*dADD1a* and lane 5) *Sgs3*-GAL4/UAS-*dADD1b. rp49* transcript was used as a control*.* Parameters of area (d) and intensity (e) of wild-type and overexpression conditions were quantified. Ordinary one-way ANOVA was performed to determine significance. No significant differences were found. For each genotype we counted the number of nuclei wt *n* = 303, *UAS-dADD1; Sgs3-GAL4 n* = 307, *Sgs3*-GAL4/UAS-*dADD1a*, *n* = 296, *Sgs3*-GAL4/UAS-*dADD1b n* = 219. The quantification was made with ImageJ.

**Additional file 3 **Overexpression of all dADD1 isoforms disturbs H3K9me3 and dADD1 signal. a) Immunostaining of polytene chromosomes from wild-type, and over-expressing dADD1 a and b proteins.*,* DNA (grey signal), HP1a (red signal), H3K9me3 (green) and Merge scale bar 20 μm. b) Immunostaining of polytene chromosomes from wild-type and over-expressing dADD1 proteins. Genotype: *Sgs3*-GAL4/UAS-*dADD1a* and *Sgs3*-GAL4/UAS-*dADD1b,* DNA (grey signal), pan-dAdd1 (green), HP1a (red signal) and Merge scale bar 20 μm.

**Additional file 4 **Schematic representation of *Drosophila* polytene chromosomes and the location of the transcripts analyzed in Fig. 5. The numbers below each chromosome correspond to cytological map locations. Genes targeted by HP1a are shown in red, genes targeted by Su (var)3–9 are shown in green and in yellow, the genes that are regulated by both proteins. Euchromatic genes are represented with blue lines. A black circle represents the chromocenter.

**Additional file 5 **Localization of dADD1a protein in the analyzed genes. dADD1a (pink peaks) is located principally at the promoter, and through the gene bodies in all the pericentric genes (*cta and lt*). Also, at *Ank* controlled by HP1a/Su (var)3–9. dADD1a is not present in euchromatic genes such as *Sgs8*, *Actin* and *Hsp70Aa* neither in *Su (var)205* and *Su (var)3–9* nor in genes controlled by Su (var)3–9 such as *Psi* and *kraken* or HPIa exclusively controlled genes as *toy* or *lgs*.

**Additional file 6 **Raw data.

## Data Availability

All data generated during this study is included in this published article. The data sets analyzed during the current study are available at the public databases mentioned in the Methods section of this article. Raw figures have been added as an Additional file 6.

## Abbreviations

ADD: A zinc finger domain named after three proteins: ATRX, DNMT3 and DNMT3L
ASF1: Anti-Silencing function 1 protein
ATRX: Alpha-thalassemia and Mental Retardation X-related
dADD1: Drosophila ADD containing domain protein 1
dXNP: Drosophila X-linked Nuclear Protein
H3K4: Lysine 4 of Histone H3
H3K9me3: Trimethylation of lysine 9 of histone H3
HP1a: Heterochromatin Protein 1 variant a
JAK: Janus Kinase
JNK: c-Jun N terminal Kinase
Pol II: RNA Polymerase II
RAD54: DNA repair and recombination protein 54
STAT92E: Signal Transducer and Activator Transcription and 92E band
