# Supplementary figures and images for: Molecular effects of dADD1 misexpression in chromatin organization and transcription
Source: BMC Mol Cell Biol. 2020 Mar 23;21:17. doi: 10.1186/s12860-020-00257-2 (PMC7092677; doi:10.1186/s12860-020-00257-2)

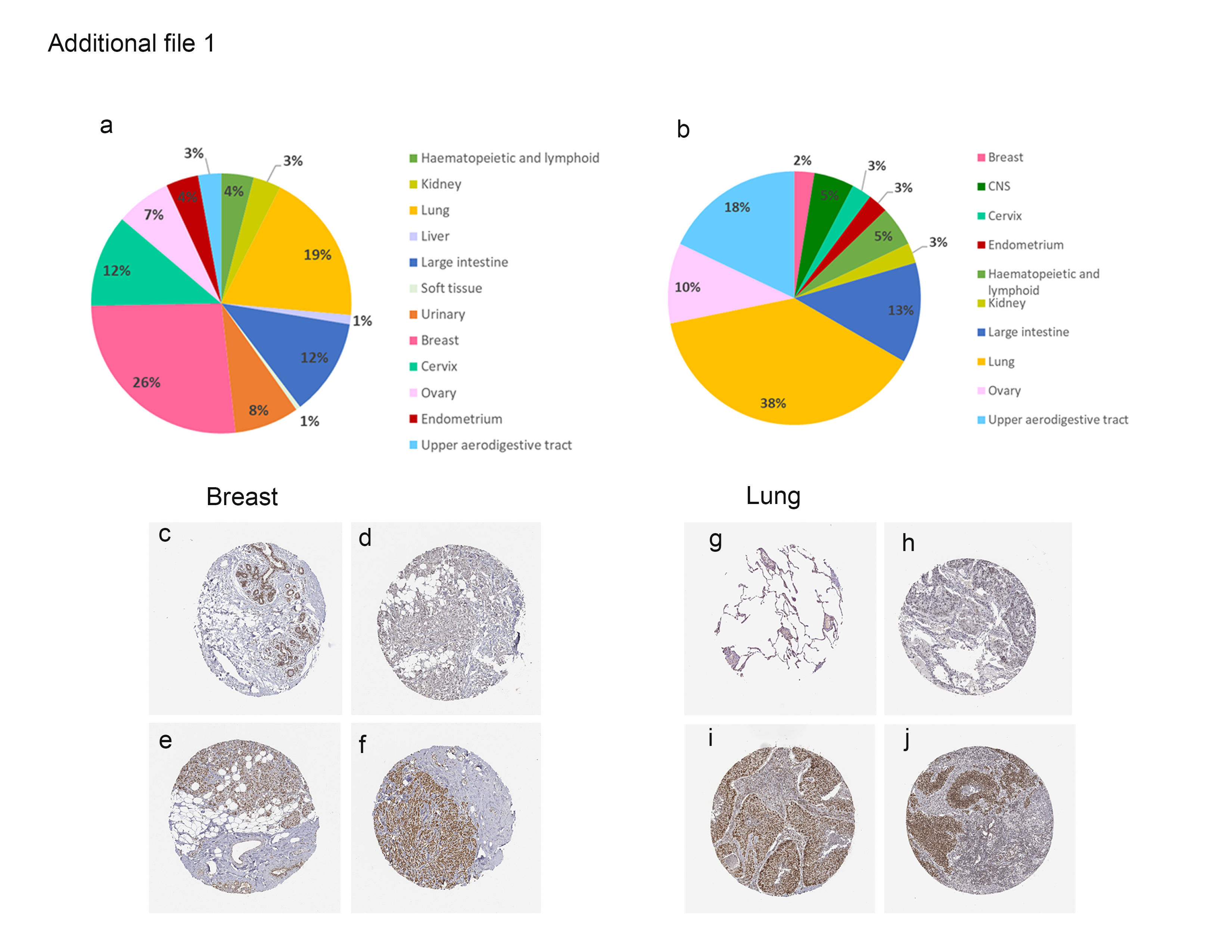

Supplement: Supplementary file 1 — Additional file 1. Distribution of cases by cancer type expressed as a percentage with a) ATRX overexpression and b) ATRX underexpression. Databases last accessed on February 18, 2019. C) Healthy breast sample tissue (Patient id: 3544) from a 45 years old female patient showing ATRX immunohistochemistry, d) Breast duct carcinoma sample (Patient id: 1874) from an 80-year-old patient. e) Breast duct carcinoma sample (Patient id: 4193) from a 43-year-old patient with a high signal of ATRX. f) Lobular carcinoma sample (Patient id: 4789) from a 49-year-old patient with a high signal of ATRX. g) Healthy Lung sample tissue (Patient id: 1678) from a 57-years-old female patient showing ATRX immunohistochemistry, h) Adenocarcinoma from female 51 years old (Patient id: 2041) showing low signal of ATRX, i) Squamous cell carcinoma from Male, 64 years old (Patient id:4090), j) Squamous cell carcinoma from Male, 72 years old (Patient id:4896) with high signal of ATRX. The immunohistochemistry was performed with the same antibody sc-15,408 from Santa Cruz Biotechnology. Image credit: Human Protein Atlas. [file 12860_2020_257_MOESM1_ESM.tif]

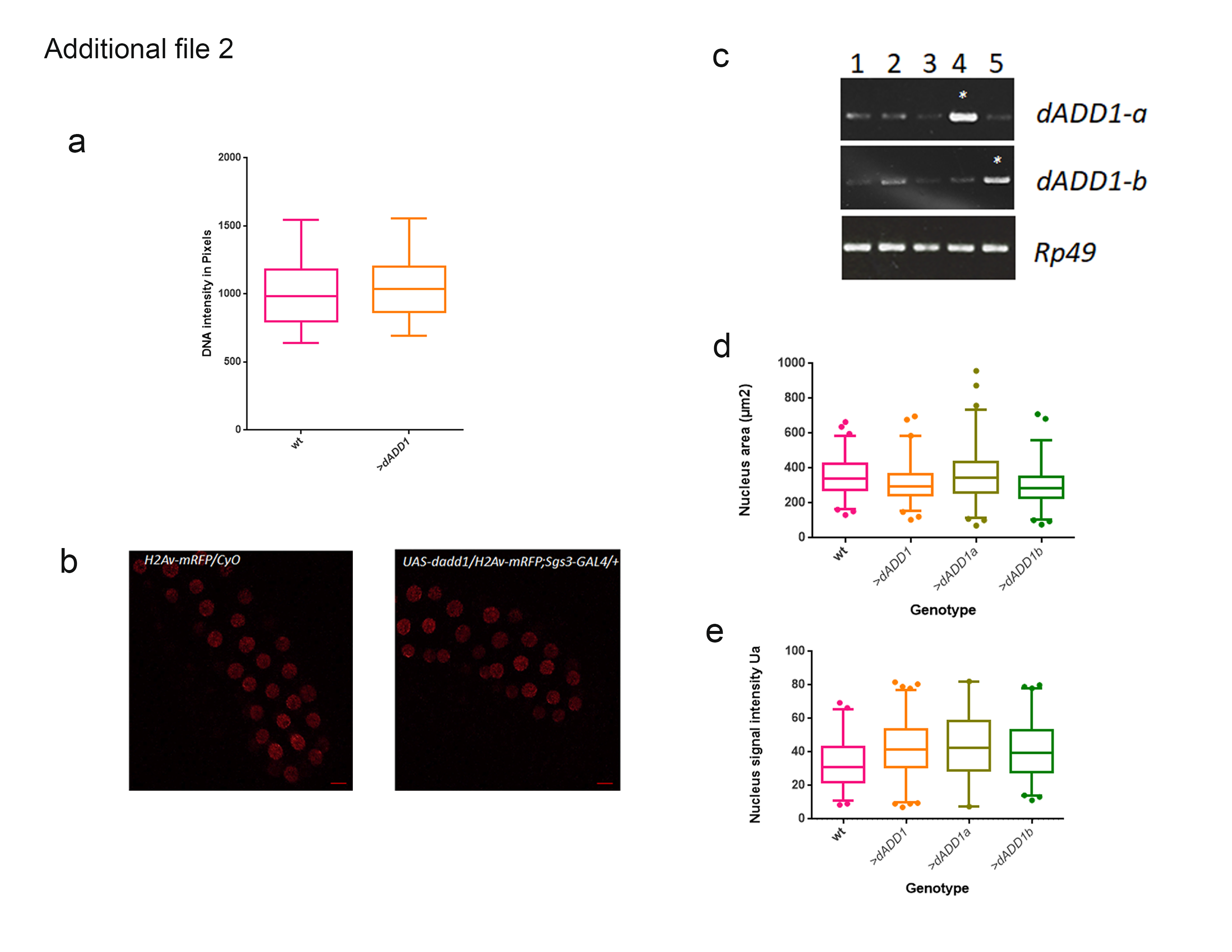

Supplement: Supplementary file 2 — Additional file 2 The amount of DNA and chromatin does not change during dADD1 overexpression. a) DNA quantification in salivary glands over-expressing all the isoforms. An unpaired t-test was performed to determine significance. No significant differences were found. b) H2Av-RFP visualization of salivary glands with an H2Av-RFP transgenic line (red signal) the amount of chromatin between wild-type and over-expression of dADD1 does not change H2Av-RFP signal intensity. c) Transcript analyzes by RT-PCR lane 1) Sgs3-GAL4, lane 2) UAS-dADD1a, lane 3) UAS-dADD1b, lane 4) Sgs3-GAL4/UAS-dADD1a and lane 5) Sgs3-GAL4/UAS-dADD1b. rp49 transcript was used as a control. Parameters of area (d) and intensity (e) of wild-type and overexpression conditions were quantified. Ordinary one-way ANOVA was performed to determine significance. No significant differences were found. For each genotype we counted the number of nuclei wt n = 303, UAS-dADD1; Sgs3-GAL4 n = 307, Sgs3-GAL4/UAS-dADD1a, n = 296, Sgs3-GAL4/UAS-dADD1b n = 219. The quantification was made with ImageJ. [file 12860_2020_257_MOESM2_ESM.tif]

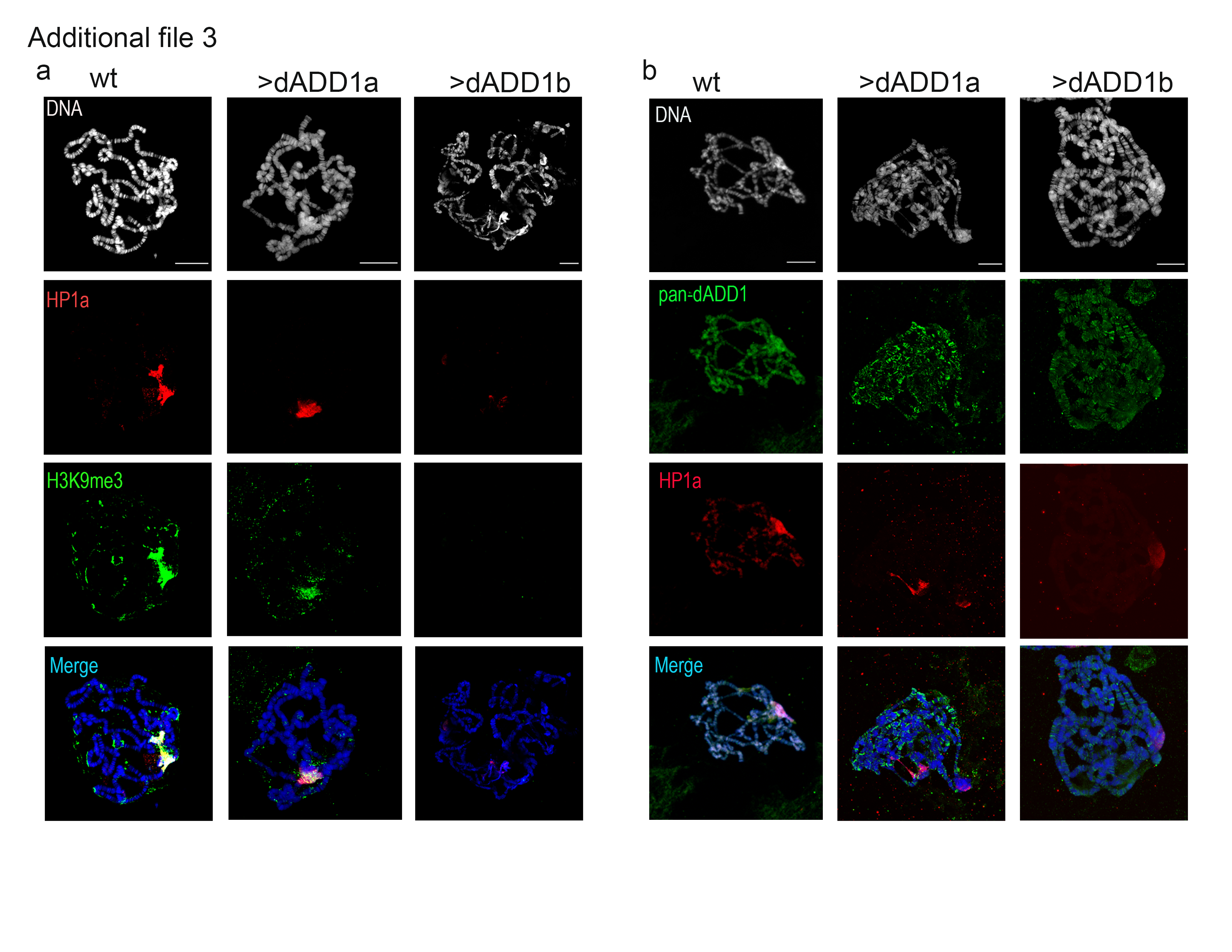

Supplement: Supplementary file 3 — Additional file 3 Overexpression of all dADD1 isoforms disturbs H3K9me3 and dADD1 signal. a) Immunostaining of polytene chromosomes from wild-type, and over-expressing dADD1 a and b proteins., DNA (grey signal), HP1a (red signal), H3K9me3 (green) and Merge scale bar 20 μm. b) Immunostaining of polytene chromosomes from wild-type and over-expressing dADD1 proteins. Genotype: Sgs3-GAL4/UAS-dADD1a and Sgs3-GAL4/UAS-dADD1b, DNA (grey signal), pan-dAdd1 (green), HP1a (red signal) and Merge scale bar 20 μm. [file 12860_2020_257_MOESM3_ESM.tif]

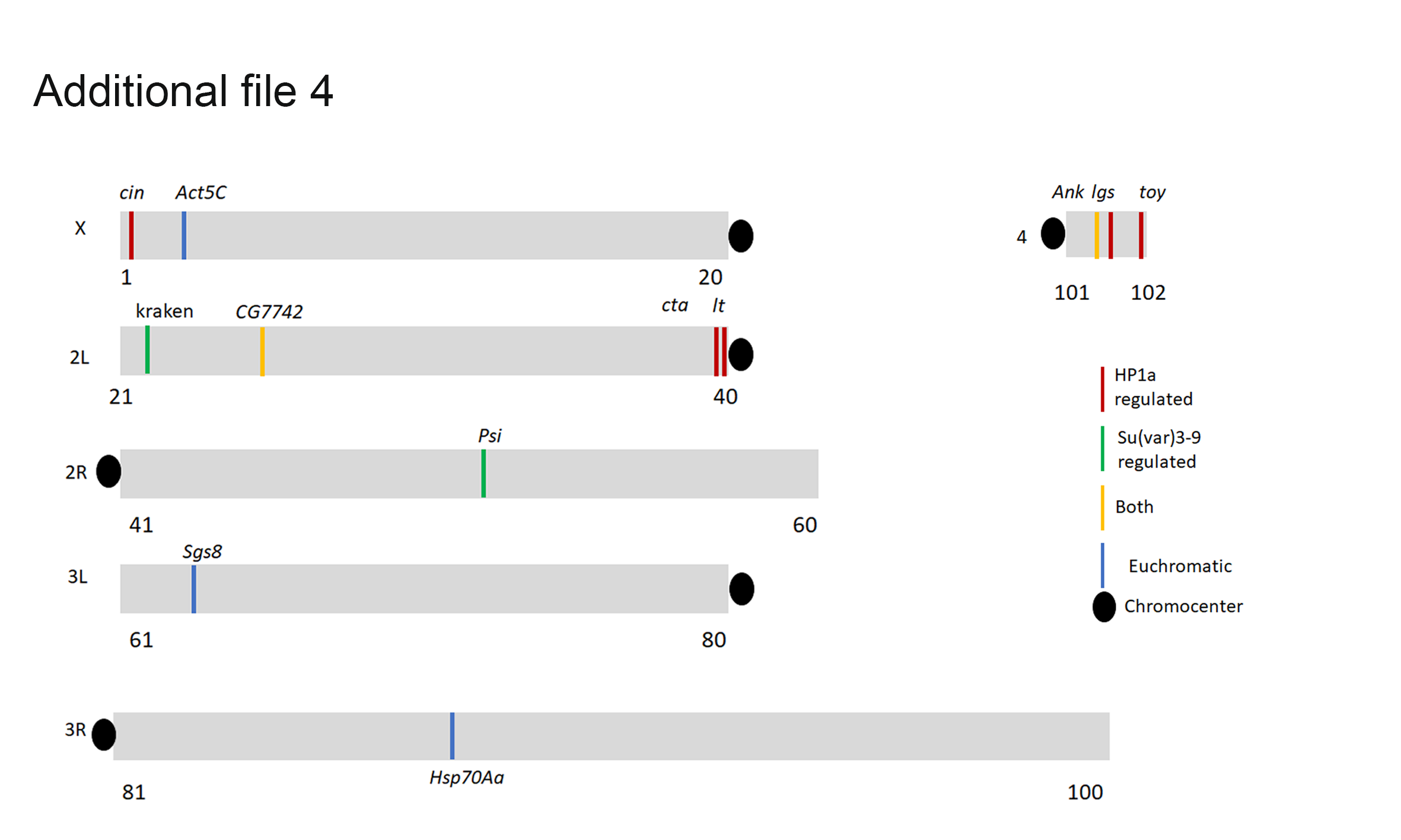

Supplement: Supplementary file 4 — Additional file 4 Schematic representation of Drosophila polytene chromosomes and the location of the transcripts analyzed in Fig. 5. The numbers below each chromosome correspond to cytological map locations. Genes targeted by HP1a are shown in red, genes targeted by Su (var)3–9 are shown in green and in yellow, the genes that are regulated by both proteins. Euchromatic genes are represented with blue lines. A black circle represents the chromocenter. [file 12860_2020_257_MOESM4_ESM.tif]

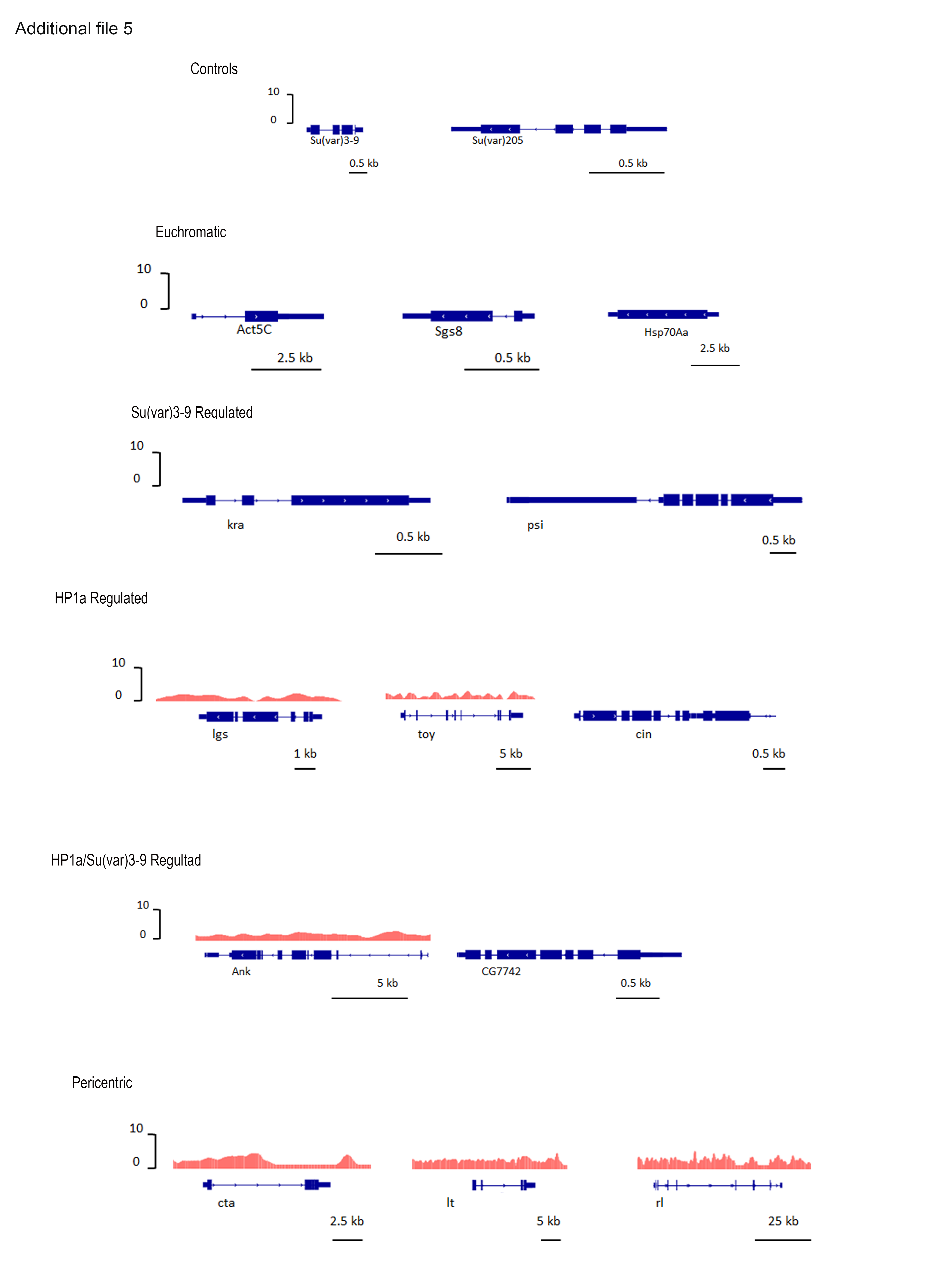

Supplement: Supplementary file 5 — Additional file 5 Localization of dADD1a protein in the analyzed genes. dADD1a (pink peaks) is located principally at the promoter, and through the gene bodies in all the pericentric genes (cta and lt). Also, at Ank controlled by HP1a/Su (var)3–9. dADD1a is not present in euchromatic genes such as Sgs8, Actin and Hsp70Aa neither in Su (var)205 and Su (var)3–9 nor in genes controlled by Su (var)3–9 such as Psi and kraken or HPIa exclusively controlled genes as toy or lgs. [file 12860_2020_257_MOESM5_ESM.tif]

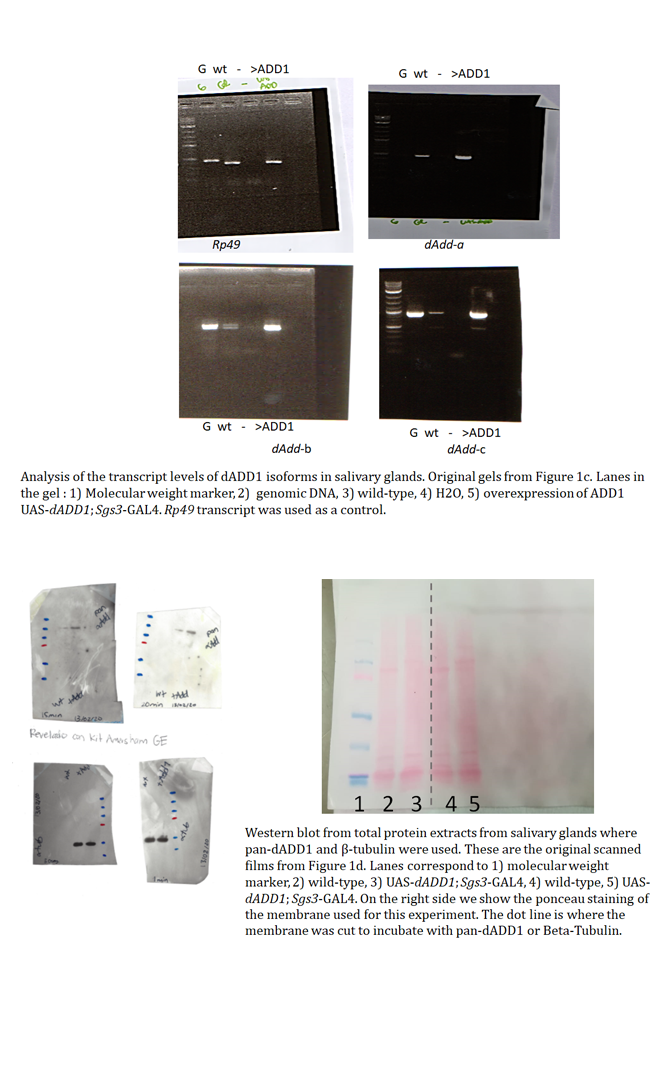

Supplement: Supplementary file 6 — Additional file 6 Raw data. [file 12860_2020_257_MOESM6_ESM.tif]
